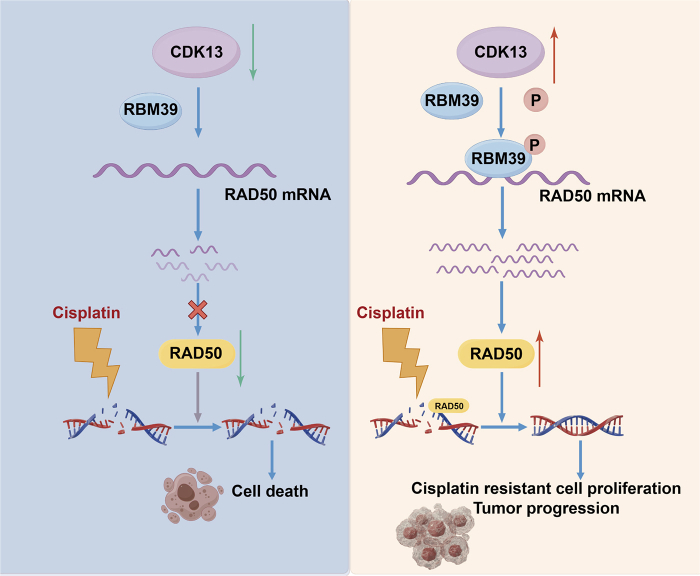# Phosphorylation of RBM39 by CDK13 stabilizes *RAD50* mRNA to drive cisplatin resistance in endometrial cancer

**DOI:** 10.1016/j.jbc.2026.111447

**Published:** 2026-04-15

**Authors:** Chenxiao Yang, Xiaoyan Zheng, Hao Sun, Juntong Du, Youjun Luo, Chang Wang, Yunlong Wu, Liying Qu, Fang Yuan, Zhan Yang

**Affiliations:** 1 Center of Tumor Immunology and Cytotherapy, Medical Research Center, The Affiliated Hospital of Qingdao University, Qingdao, Shandong, China; 2 Department of Obstetrics and Gynecology, The Affiliated Hospital of Qingdao University, Qingdao, Shandong, China; 3 Department of Obstetrics and Gynecology, The Fifth Affiliated Hospital of Southern Medical University, Guangzhou, Guangdong, China; 4 Department of Anesthesiology, The Affiliated Hospital of Qingdao University, Qingdao, Shandong, China

**Keywords:** cisplatin resistance, CDK13, endometrial cancer, DNA damage repair, RBM39

## Abstract

Platinum-resistant is a major therapeutic challenge in advanced endometrial cancer (EC), often driven by enhanced DNA damage repair, yet the underlying molecular mechanisms remain incompletely understood. Here, we report that cyclin-dependent kinase 13 (CDK13) is significantly overexpressed in EC tissues, where its high expression correlates with poor patient survival and clinical resistance to platinum-based chemotherapy. Functionally, CDK13 overexpression promoted EC cell proliferation and conferred cisplatin resistance both *in vitro* and *in vivo*, whereas its knockdown potentiated cisplatin-induced apoptosis and DNA damage. Mechanistically, through phosphoproteomic analysis, we identified the RNA-binding protein RNA-binding motif protein 39 (RBM39) as a critical downstream target of CDK13. We demonstrate that CDK13 directly phosphorylates RBM39 at serine 117, and this phosphorylation is essential for CDK13-mediated resistance. Crucially, phosphorylation at Ser117 enhanced the ability of RBM39 to bind and stabilize the mRNA of *RAD50*, a key DNA repair gene. Post-transcriptional regulation led to an increase in RAD50 protein, which facilitated the repair of DNA damage caused by cisplatin, promoting the survival of cells. The therapeutic relevance of this axis was confirmed *in vivo*, where dual knockdown of RBM39 and RAD50 synergistically sensitized EC xenografts to cisplatin. Our study first unveils a novel CDK13/RBM39/RAD50 signaling axis that drives platinum resistance in EC by enhancing DNA damage repair *via* mRNA stabilization, revealing promising therapeutic targets for overcoming chemoresistance in this malignancy.

Endometrial cancer (EC) is a prevalent gynecologic malignancy whose incidence is rising globally, including among younger women (1, 2). Uterine corpus endometrial carcinoma, the most common histological subtype, carries a grim prognosis for advanced or recurrent cases, with a 5-year survival rate of only ∼17% (3, 4). Clinical management often combines surgery with platinum-based chemotherapy, wherein cisplatin serves as a first-line treatment for advanced disease (5, 6). However, acquired resistance significantly hampers its efficacy, contributing to disease progression and mortality (7). Enhanced DNA damage repair (DDR) is a key mechanism underlying this resistance (8); therefore, elucidating the molecular basis of cisplatin resistance and developing strategies to counteract it are urgent priorities in EC research.

Cyclin-dependent kinases (CDKs) are broadly categorized into cell cycle regulators (*e*.*g*., CDK1-4, CDK6) and transcriptional regulators (*e*.*g*., CDK7-9, CDK12, CDK13) (9). Beyond their canonical roles, dysregulation of specific CDKs is increasingly implicated in chemotherapy resistance. For instance, CDK12 alterations are linked to PARP inhibitor response in prostate cancer (10), while CDK7/9 inhibition sensitizes cancer cells by disrupting transcriptional programs essential for survival and DDR (11). CDK13, also known as CDC2L5, is an emerging transcriptional CDK that phosphorylates RNA polymerase II and regulates transcription elongation and RNA splicing (12). Accumulating evidence underscores its oncogenic roles across various malignancies. Recent studies highlight its role in modulating DDR and antitumor immunity (13). Our prior work revealed that CDK13 promotes prostate cancer progression *via* NSUN5-mediated *ACC1* mRNA modification and forms a feedback loop with *circCDK13* (14, 15). Despite these advances, the function of CDK13 in EC—particularly its potential role in chemotherapy resistance—remains largely unexplored.

The RNA-binding motif protein 39 (RBM39) functions as a pre-mRNA splicing factor and transcriptional coactivator (16). Beyond these roles, RBM39 is increasingly implicated in oncogenesis and therapy resistance. In liver cancer, it drives metabolic reprogramming by enhancing metabolism-related gene expression (17). In viral latency, it scaffolds an m^6^A-dependent RNA decay complex (18), and in renal cell carcinoma, targeting RBM39 with Tasisulam enhances TRAIL-induced apoptosis (19). Collectively, emerging evidence positions RBM39 as a pivotal regulator in oncogenesis and therapy response through its roles in RNA metabolism and genome stability (16, 20). However, whether RBM39 contributes to chemotherapy resistance in EC remains unknown.

RAD50 is a core component of the MRE11-RAD50-NBS1 complex, which is essential for DNA double-strand break repair (21). Its upregulation in various cancers is closely associated with platinum resistance, serving as an independent prognostic indicator in colorectal and ovarian cancers (22, 23). Mechanistically, RAD50 counteracts cisplatin cytotoxicity by enhancing repair of chemotherapy-induced DNA lesions (23, 24). Critically, whether RBM39 regulates RAD50 expression and how this regulatory axis contributes to cisplatin resistance in EC has never been investigated.

In this study, we demonstrate that CDK13 is significantly overexpressed in endometrial carcinoma and is associated with poor prognosis and platinum resistance. Our results further reveal that CDK13 phosphorylates RBM39 at Ser117, which in turn enhances the mRNA stability of *RAD50*, a key DNA damage repair gene. This CDK13/RBM39/RAD50 signaling axis facilitates the repair of cisplatin-induced DNA damage, thereby promoting chemoresistance.

## Results

### CDK13 is overexpressed in EC and correlates with platinum resistance

**Figure 1:**
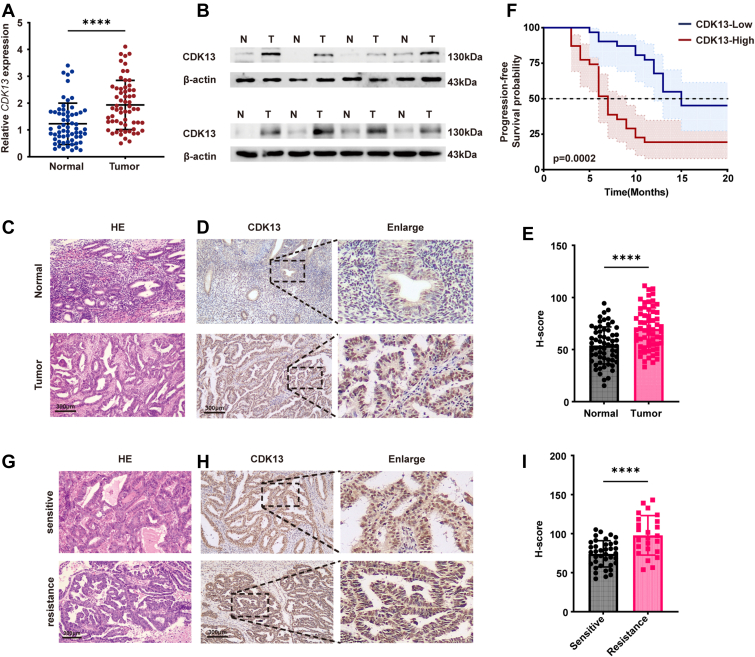
**CDK13 is highly expressed and associated with platinum resistance**. *A*, *CDK13* mRNA expression levels in normal endometrial(n = 62) and EC tissues(n = 62) were evaluated by RT-qPCR. Data are presented as mean ± SD, with individual data points superimposed. Statistical analysis was performed using unpaired two-tailed Student's *t* test. ∗∗∗∗*p*< 0.0001. *B*, Western blot analysis of CDK13 protein levels in normal endometrial and EC tissues. *C*, representative H&E staining of EC and adjacent non-tumor endometrial tissues. The scale bar represents = 300 μm. *D* and *E*, representative IHC images showing CDK13 protein expression in EC and non-tumor endometrial tissues (n = 62). The scale bar represents = 300 μm. H-score analysis of IHC is shown in (*E*). Data are presented as mean ± SD, with individual data points superimposed. Statistical analysis was performed using unpaired two-tailed Student's *t* test. ∗∗∗∗*p*< 0.0001. *F*, Kaplan-Meier survival analysis of progression-free survival in 62 EC patients stratified by CDK13 expression. Patients were divided into CDK13-high (n = 33) and CDK13-low (n = 29) groups based on the median IHC H-score. The log-rank test was used to compare survival curves. *p*< 0.05 was considered statistically significant. *G*, H&E staining of platinum-sensitive and platinum-resistant endometrial cancer tissues. The scale bar represents = 300 μm. *H* and *I*, representative IHC images of CDK13 expression in platinum-sensitive (n = 38) and platinum-resistant endometrial cancer tissues (n = 24). The scale bar represents = 300 μm. H-score analysis of IHC is shown in (*I*). Data are presented as mean ± SD, with individual data points superimposed. Statistical analysis was performed using unpaired two-tailed Student's *t* test. ∗∗∗∗*p*< 0.0001. EC, endometrial cancer; IHC, immunohistochemical.

**Supplementary Figure 1:**
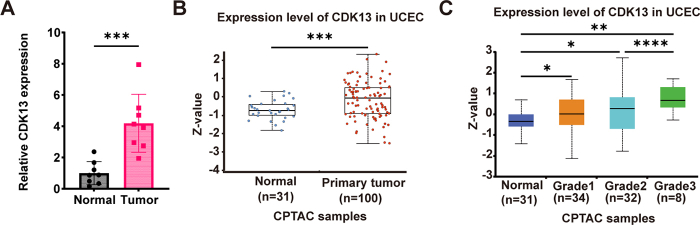


Cyclin-dependent kinase 13 (CDK13) has been implicated as an oncogenic driver in prostate and renal cell carcinomas (14, 15, 25), yet its function in EC remains unexplored. Given the critical role of platinum resistance in EC prognosis, we sought to investigate whether CDK13 contributes to this chemoresistant phenotype. To investigate its role in EC, we analyzed 62 human EC tissues alongside 62 normal endometrial tissues. RT-qPCR showed that *CDK13* mRNA levels were significantly higher in EC tissues than in normal controls (Fig. 1*A*). Consistent with this, Western blot analysis on eight randomly selected paired clinical samples confirmed a marked increase in CDK13 protein levels in tumor tissues (Figs. 1*B*, and S1*A*). Furthermore, immunohistochemical (IHC) staining demonstrated substantially higher CDK13 protein expression *in situ* within EC tissues compared to normal endometrium (Fig. 1, *C*–*E*). Moreover, we investigated the relationship between CDK13 expression and clinicopathological attributes. A higher expression level of CDK13 was associated with higher histologic grade and clinical T stage in EC patients (Table S1). Bioinformatic analysis using the UALCAN database corroborated these findings, showing significantly higher CDK13 protein levels in EC tissues, with even greater expression observed in high-grade tumors (Fig. S1, *B* and *C*). Crucially, Kaplan-Meier survival analysis stratified by the median IHC H-score revealed that patients with high CDK13 expression (n = 31) experienced a significantly shorter median progression-free survival compared to those with low expression (n = 31) (Fig. 1*F*). This correlation with poor prognosis prompted us to hypothesize a role for CDK13 in platinum-based chemotherapy resistance, a major determinant of progression-free survival in advanced EC. To test this, we compared CDK13 expression in tumors from patients clinically stratified as platinum-sensitive *versus* platinum-resistant. Remarkably, CDK13 levels were significantly higher in the resistant group (Fig. 1, *G–I*). These collective clinical findings provided a strong rationale to functionally dissect the role of CDK13 in regulating platinum sensitivity in EC.

### CDK13 promotes EC cell proliferation and confers cisplatin resistance *in vitro* and *in vivo*

**Figure 2:**
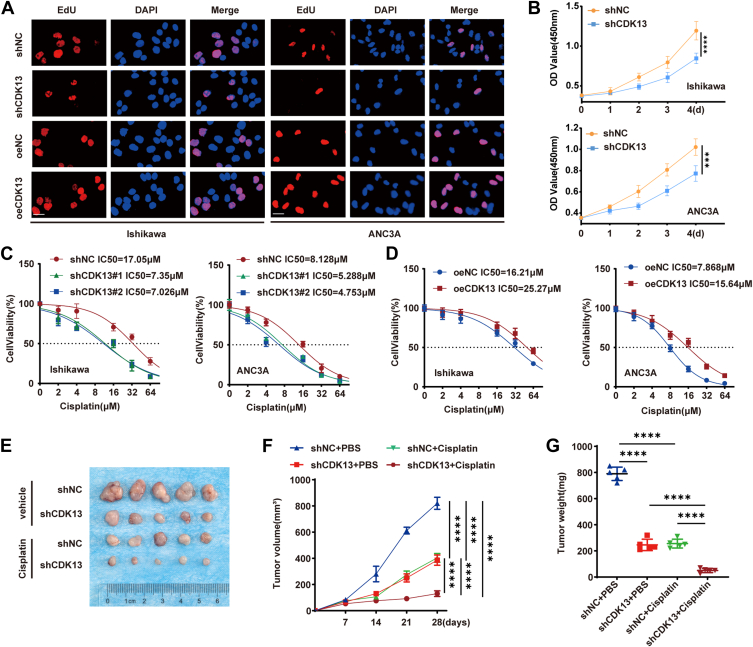
**CDK13 promotes proliferation and cisplatin resistance in EC cells *in vitro* and *in vivo***. *A*, cell proliferation was evaluated by EdU incorporation assay in CDK13-manipulated EC cells. Representative images, The scale bar represents = 20 μm. *B*, cell proliferation was assessed by CCK-8 assay in CDK13-knockdown EC cells. Data are presented as mean ± SD from three independent experiments, with individual data points superimposed. Statistical analysis was performed using two-way ANOVA with Tukey's *post hoc* test for multiple comparisons. ∗*p*< 0.05, ∗∗*p*< 0.01, ∗∗∗*p*< 0.001 *versus* corresponding control. *C* and *D*, cell viability of CDK13-manipulated EC cells treated with increasing concentrations of cisplatin for 48 h was measured by CCK-8 assay. IC50 values are indicated. Data are presented as mean ± SD from three independent experiments, with individual data points superimposed. Statistical analysis was performed using one-way ANOVA with Tukey's *post hoc* test for multiple comparisons. ∗*p*< 0.05, ∗∗*p*< 0.01, ∗∗∗*p*< 0.001. *E* and *G*, xenograft tumor models were established in BALB/c nude mice (n = 8 per group) by injecting control (shNC) or CDK13-knockdown (shCDK13) cells, followed by treatment with or without cisplatin. *E*, representative photographs of resected xenograft tumors from each group at the endpoint. *F*, tumor growth curves and (*G*) final tumor weights are shown. Data are presented as mean ± SD, with individual data points superimposed. Tumor growth curves were analyzed using two-way repeated-measures ANOVA followed by Tukey's *post hoc* test. Tumor weights were analyzed using one-way ANOVA with Tukey's *post hoc* test for multiple comparisons, with data presented as mean ± SD, with individual data points superimposed. ∗*p*< 0.05, ∗∗*p*< 0.01, ∗∗∗*p*< 0.001. EC, endometrial cancer; IHC, immunohistochemical.

**Supplementary Figure 2:**
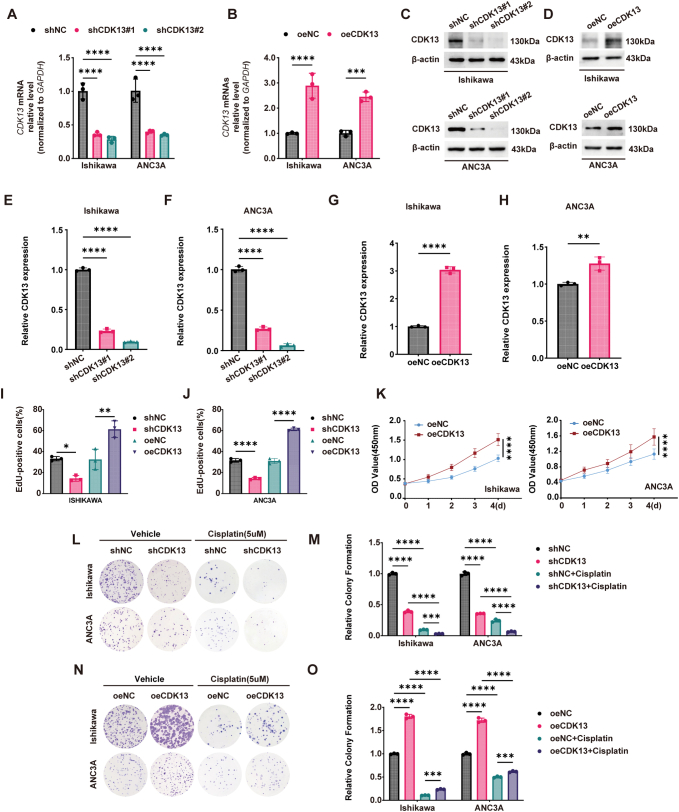


To elucidate the functional role of CDK13 in EC, we performed CDK13 knockdown and overexpression in two EC cell lines, Ishikawa and AN3CA. Efficient modulation of *CDK13* expression was confirmed at both the mRNA level by RT-qPCR (Fig. S2, *A* and *B*) and the protein level by Western blot analysis (Fig. S2, *C–H*). Assessment of proliferative capacity using 5-Ethynyl-2'-deoxyuridine (EdU) incorporation assays showed that CDK13 knockdown significantly reduced the percentage of EdU-positive cells, whereas CDK13 overexpression enhanced it (Figs. 2*A*, and S2, *I* and *J*). Supporting these findings, CCK-8 assays demonstrated that CDK13 knockdown inhibited cell proliferation, while its overexpression promoted it (Figs. 2*B* and S2*K*), indicating a pro-proliferative role for CDK13 in EC cells. Given that platinum agents are first-line chemotherapy for EC (5, 6), we evaluated the impact of CDK13 on cisplatin sensitivity. Clonogenic assays revealed that CDK13 knockdown suppressed colony formation, an effect potentiated by cisplatin treatment. Conversely, CDK13 overexpression enhanced colony formation and partially rescued the cisplatin-induced suppression (Fig. S2, *L–O*). Dose-response curves measuring cell viability showed that CDK13 overexpression increased the IC50 value for cisplatin, whereas knockdown decreased it (Fig. 2, *C* and *D*), indicating that CDK13 depletion sensitizes EC cells to cisplatin. To validate these findings *in vivo*, we established xenograft tumor models in nude mice using control or stable CDK13-knockdown cells. Cisplatin treatment significantly inhibited tumor growth, and this inhibitory effect was further enhanced by CDK13 knockdown (Fig. 2, *E* and *F*). Tumor weight measurements at the endpoint yielded consistent results (Fig. 2*G*). Together, CDK13 knockdown suppresses cell proliferation and enhances the sensitivity of EC cells to platinum drugs both *in vitro* and *in vivo*.

### CDK13 facilitates the repair of cisplatin-induced DNA damage

**Figure 3:**
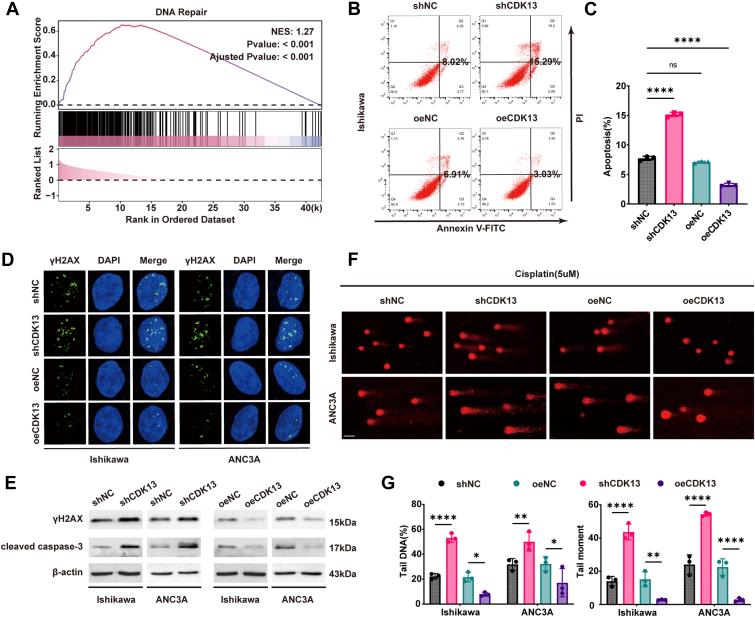
**CDK13 facilitates the repair of cisplatin-induced DNA damage**. *A*, gene Set Enrichment Analysis plot showing the association between CDK13 expression and the DNA damage repair pathway in EC (*p*< 0.001). *B* and *C*, apoptosis was analyzed by flow cytometry in CDK13-manipulated Ishikawa cells treated with or without cisplatin (5 μM) for 48 h. Representative plots (*B*) and quantification (*C*) are shown. Data are presented as mean ± SD from three independent experiments, with individual data points superimposed. Statistical analysis was performed using one-way ANOVA with Tukey's *post hoc* test for multiple comparisons. ∗*p*< 0.05, ∗∗*p*< 0.01, ∗∗∗*p*< 0.001. *D*, immunofluorescence analysis of γH2AX foci (*green*) in EC cells treated with cisplatin (5 μM) for 24 h. Nuclei were counterstained with DAPI (*blue*). *E*, Western blot analysis of γH2AX and cleaved caspase-3 protein levels in CDK13-manipulated EC cells treated with cisplatin (5 μM) for 48 h *F* and *G*, representative images (*F*) and quantification (*G*) of the alkaline comet assay performed in control, CDK13-overexpressing, and CDK13-knockdown EC cells treated with cisplatin (5 μM) for 48 h. The scale bar represents = 20 μm. Data are presented as mean ± SD from three independent experiments, with individual data points superimposed. Statistical analysis was performed using one-way ANOVA with Tukey's *post hoc* test for multiple comparisons. ∗∗*p*< 0.01, ∗∗∗*p*< 0.001. EC, endometrial cancer.

**Supplementary Figure 3:**
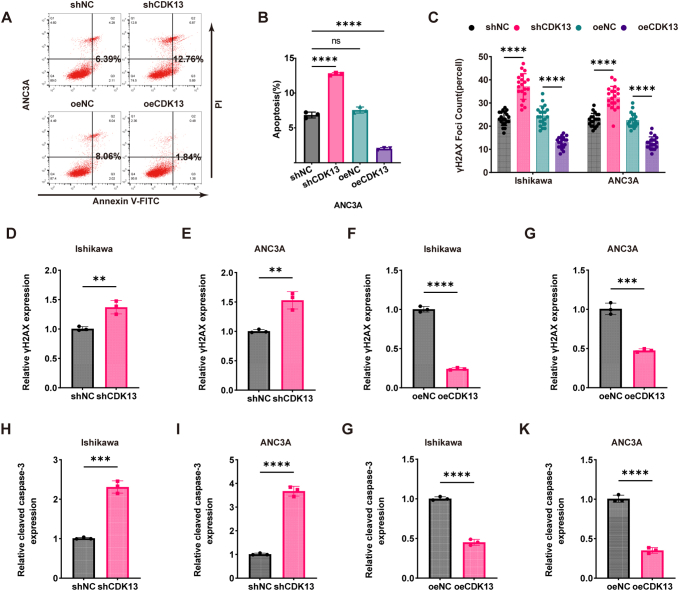


Platinum drugs primarily exert their cytotoxic effects by forming DNA adducts, interfering with DNA replication and repair, ultimately inducing apoptosis (26, 27). Enhanced DNA damage repair capacity in tumor cells is a key mechanism underlying platinum resistance (28, 22). To determine if CDK13 is involved in platinum resistance by affecting DNA damage, we analyzed TCGA data using Gene Set Enrichment Analysis, which showed a notable connection between *CDK13* expression and DNA damage repair pathways (Fig. 3*A*). We first assessed the role of CDK13 in cisplatin-induced cell death using flow cytometry. CDK13 knockdown potentiated cisplatin-induced apoptosis, whereas CDK13 overexpression attenuated it (Figs. 3, *B* and *C*, and S3, *A* and *B*). To assess the functional outcome of cisplatin-induced DNA damage, which culminates in the formation of double-strand breaks, we performed immunofluorescence staining for γH2AX, a hallmark of DSBs. Immunofluorescence staining for the DNA double-strand break marker γH2AX indicated that reducing CDK13 levels worsened DNA damage caused by cisplatin, whereas increasing CDK13 levels reduced the damage (Figs. 3*D*, and S3*C*). Western blot analysis further confirmed that CDK13 knockdown upregulated levels of γH2AX and the apoptosis marker cleaved caspase-3, whereas CDK13 overexpression suppressed their induction by cisplatin (Figs. 3*E*, and S3, *D–K*). Additionally, alkaline comet assays demonstrated that CDK13 knockdown significantly increased cisplatin-induced comet tail moment and tail DNA percentage, indicative of greater DNA damage. Overexpression of CDK13 reduced these parameters (Fig. 3, *F* and *G*). These results collectively indicate that CDK13 protects against cisplatin-induced DNA damage, thereby reducing cisplatin sensitivity in EC cells.

### RAD50 mediates CDK13-induced cisplatin resistance in EC cells

**Figure 4:**
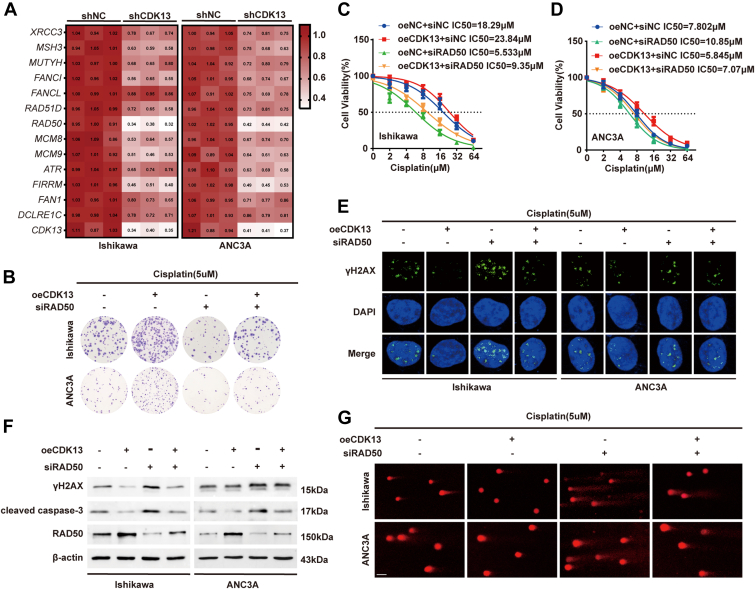
**CDK13 regulates cisplatin resistance through RAD50-mediated DNA damage repair in EC**. *A*, relative mRNA expression of indicated DNA damage repair-related genes in Ishikawa and AN3CA cells transfected with shCDK13 or control vector, measured by RT-qPCR. *B*, colony formation assays were performed in EC cells co-transfected with oeCDK13 and/or siRAD50. *C* and *D*, IC50 values for cisplatin were determined by CCK-8 assay in Ishikawa (*C*) and AN3CA (*D*) cells co-transfected with oeCDK13 and/or siRAD50. Data are presented as mean ± SD from three independent experiments, with individual data points superimposed. Statistical analysis was performed using one-way ANOVA with Tukey's *post hoc* test for multiple comparisons. ∗*p*< 0.05, ∗∗*p*< 0.01, ∗∗∗*p*< 0.001. *E*, IF analysis of γH2AX foci (*green*) in the indicated EC cells treated with cisplatin (5 μM) for 24 h. Nuclei were stained with DAPI (*blue*). *F*, Western blot analysis of RAD50, γH2AX, and cleaved caspase-3 protein levels in the indicated EC cells treated with cisplatin (5 μM) for 48 h. *G*, representative images of the alkaline comet assay performed in the indicated EC cells treated with cisplatin (5 μM) for 48 h. The scale bar represents = 20 μm. EC, endometrial cancer.

**Supplementary Figure 4:**
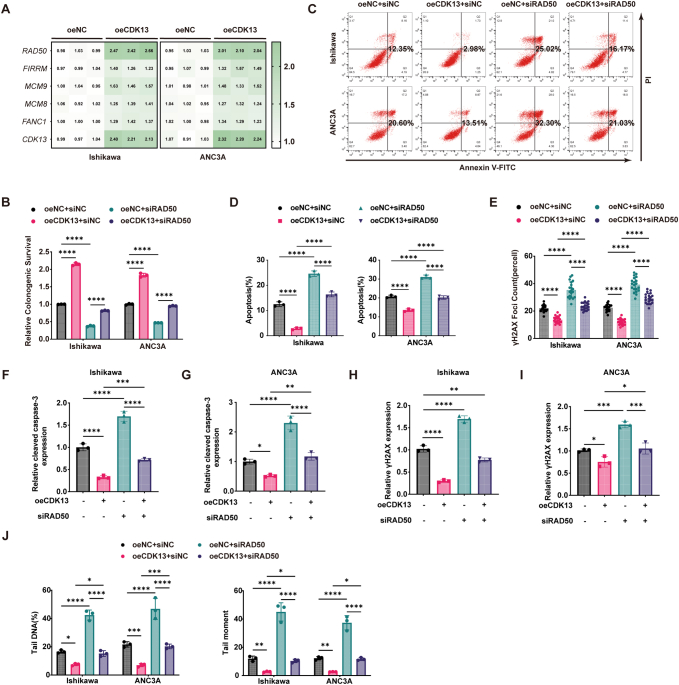


To identify key downstream effectors of CDK13 within the DNA damage repair pathway, we systematically screened for genes whose expression correlated positively with *CDK13*. Among the candidates, *RAD50* and *FIRRM* mRNA levels were significantly downregulated upon CDK13 knockdown in both Ishikawa and AN3CA cells (Fig. 4*A*). Subsequent validation in both cell lines identified *RAD50* as the most consistently and significantly upregulated gene upon CDK13 overexpression (Fig. S4*A*), prompting its selection for further study. We next investigated whether CDK13 promotes EC progression and cisplatin resistance through RAD50. Clonogenic assays showed that RAD50 knockdown reversed the pro-proliferative effect conferred by CDK13 overexpression (Figs. 4*B*, and S4*B*). The IC50 values for cisplatin indicated that the rise in IC50 caused by CDK13 overexpression was somewhat counteracted by simultaneous RAD50 knockdown (Fig. 4, *C* and *D*). Flow cytometric analysis demonstrated that RAD50 knockdown partially counteracted the anti-apoptotic effect of CDK13 overexpression under cisplatin treatment (Fig. S4, *C* and *D*). IF staining for γH2AX showed that RAD50 knockdown partially restored the DNA damage suppressed by CDK13 overexpression (Figs. 4*E*, and S4*E*). Western blot analysis confirmed that CDK13 overexpression upregulated RAD50 protein levels and concurrently downregulated γH2AX and cleaved caspase-3. Co-knockdown of RAD50 partially reversed the CDK13-mediated suppression of these DNA damage and apoptosis markers (Figs. 4*F*, and S4, *F–I*). Similarly, alkaline comet assays demonstrated that knocking down RAD50 heightened DNA damage from cisplatin (comet tail moment and tail DNA percentage), and this was partially counteracted by the concurrent overexpression of CDK13 (Figs. 4*G*, and S4*J*). Together, these findings show that CDK13 boosts platinum resistance in EC cells by promoting RAD50 expression, which reduces cisplatin-triggered DNA damage.

### RBM39 is a downstream target of CDK13 and its phosphorylation at Ser117 is critical for CDK13-mediated resistance

**Figure 5:**
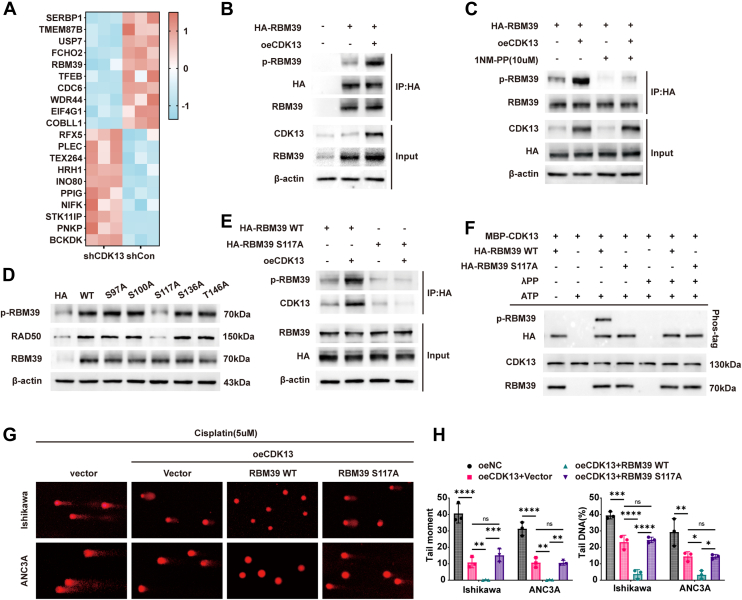
**CDK13 phosphorylates RBM39 at Ser117 to promote cisplatin resistance in EC**. *A*, heatmap of phosphorylation changes in Ishikawa cells transfected with shCDK13 *versus* shNC (*red*: *up*-regulated; *blue*: *down*-regulated). Only phosphopeptides with |log_2_FC| ≥ 0.26 (FC ≥ 1.2) and *p*< 0.05 are shown (n = 3 per group). *B*, co-IP and Western blot analysis of RBM39 phosphorylation in EC cells transfected with HA-RBM39 and/or Flag-CDK13. *C*, co-IP and Western blot analysis of RBM39 phosphorylation in Ishikawa cells transfected as indicated and treated with or without the kinase inhibitor 1 NM-PP1 (10 μM). *D*, Western blot analysis of RBM39, phospho-RBM39, and RAD50 protein levels in Ishikawa cells transfected with vectors encoding WT or phospho-deficient RBM39 mutants. *E*, co-IP and Western blot analysis assessing the interaction between CDK13 and RBM39 (WT or S117A mutant), and RBM39 phosphorylation. *F*, *In vitro* kinase assay. Purified MBP-CDK13/cyclin K was incubated with HA-RBM39 (WT or S117A) in kinase buffer with ATP and/or λPP as indicated. Phosphorylation was assessed by Phos-tag SDS-PAGE (anti-HA). *G* and *H*, representative images (*G*) and quantification (*H*) of the alkaline comet assay performed in the indicated EC cells treated with cisplatin (5 μM) for 48 h. The scale bar represents= 20 μm. Data are presented as mean ± SD from three independent experiments, with individual data points superimposed. Statistical analysis was performed using one-way ANOVA with Tukey's *post hoc* test for multiple comparisons. ∗∗*p*< 0.01, ∗∗∗*p*< 0.001. EC, endometrial cancer.

**Supplementary Figure 5:**
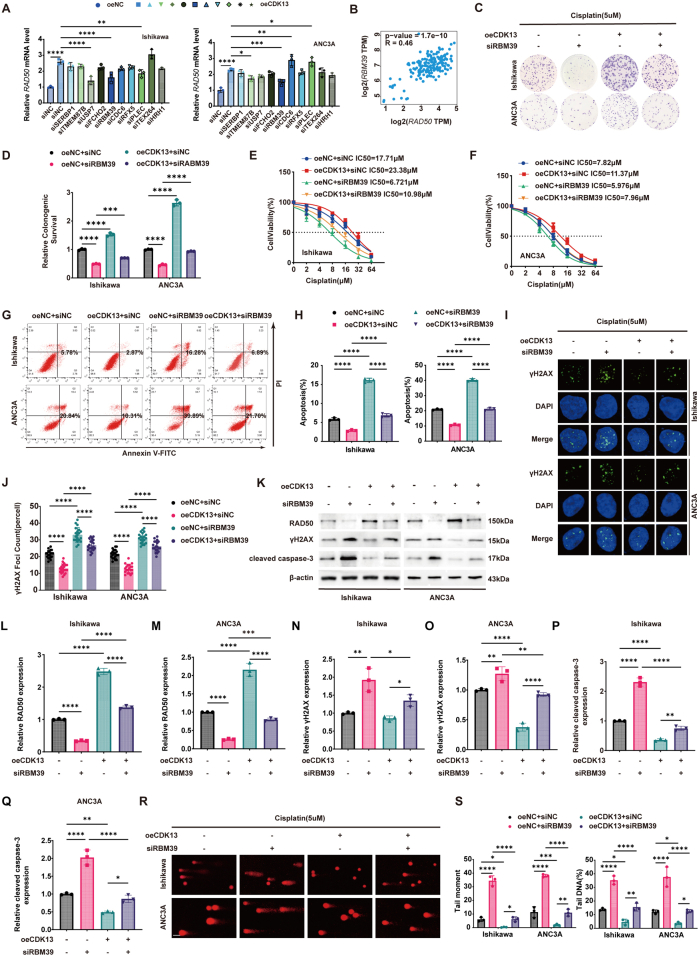


Although CDK13 upregulated *RAD50* mRNA and protein, as a cyclin-dependent kinase, it likely functions by phosphorylating downstream targets (14). To identify these targets, we performed a phosphoproteomic analysis in Ishikawa cells following CDK13 knockdown. This screen identified several proteins with reduced phosphorylation, including RBM39 (Fig. 5*A*). To determine which of these potential targets mediates CDK13's regulation of RAD50, we knocked down each candidate while overexpressing CDK13. RT-qPCR analysis revealed that only RBM39 knockdown significantly attenuated the CDK13-induced upregulation of *RAD50* mRNA in both cell lines (Fig. S5*A*), suggesting RBM39 acts upstream of RAD50. In support of this, analysis using the GEPIA database revealed a positive correlation between the expression of *RBM39* and *RAD50* (Fig. S5*B*). Functional assays confirmed the involvement of RBM39 in CDK13-mediated cisplatin resistance. Clonogenic formation assays showed that RBM39 knockdown sensitized cells to cisplatin and partially reversed the resistance induced by CDK13 overexpression (Fig. S5, *C* and *D*). Cell viability assays yielded consistent results (Fig. S5, *E* and *F*). Flow cytometry and IF staining further demonstrated that RBM39 knockdown increased apoptosis and exacerbated DNA damage, counteracting the protective effects of CDK13 (Fig. S5, *G–J*). Western blot analysis showed that RBM39 knockdown downregulated RAD50 and upregulated γH2AX and cleaved caspase-3, partially reversing the effects of CDK13 overexpression (Fig. S5, *K–Q*). Alkaline comet assays confirmed that RBM39 knockdown increased DNA damage and abrogated the reduction in damage seen with CDK13 overexpression (Fig. S5, *R* and *S*). These results establish RBM39 as a key downstream target of CDK13.

**Supplementary Figure 6:**
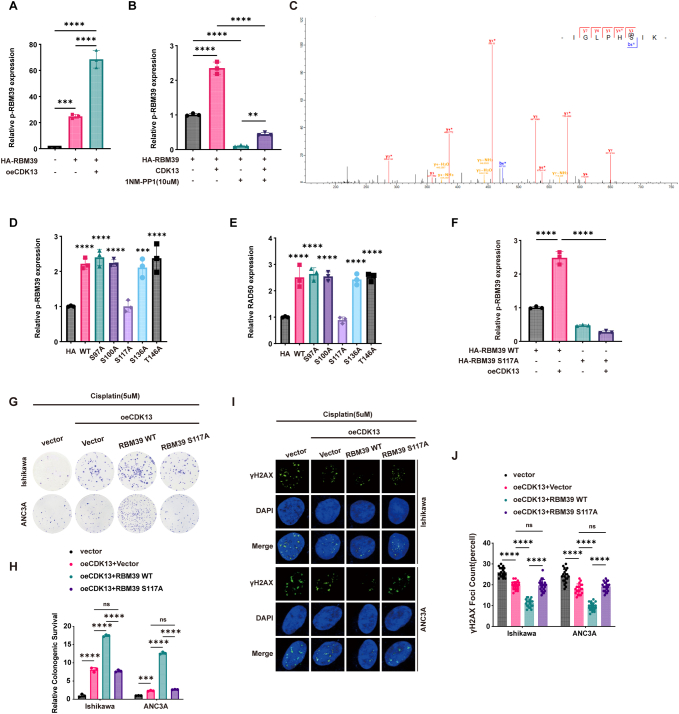


To investigate how CDK13 phosphorylates RBM39, we introduced HA-RBM39 and Flag-CDK13 plasmids into EC cells simultaneously. Co-immunoprecipitation (Co-IP) using an HA antibody showed that CDK13 overexpression enhanced RBM39 phosphorylation (Figs. 5*B*, and S6*A*). This phosphorylation was significantly inhibited when cells were treated with the Src family kinase inhibitor 1 (1NM-PP1), which also attenuated the effect of CDK13 overexpression (Figs. 5*C*, and S6*B*), suggesting kinase activity is involved. Mass spectrometric analysis of Ishikawa cells following CDK13 knockdown revealed a significant reduction in phosphorylation at the S117 residue of RBM39 (Fig. S6*C*). Based on Biogrid predictions of potential phosphorylation sites (Ser97, Ser100, Ser117, Ser136, Thr146), we generated phospho-deficient RBM39 mutants. Among these, the S117A mutation markedly reduced RBM39 phosphorylation levels (Figs. 5*D*, and S6, *D* and *E*). Co-IP experiments further demonstrated that CDK13 interacted strongly with wild-type (WT) RBM39 and promoted its phosphorylation, whereas the S117A mutation impaired this interaction and abolished CDK13-induced phosphorylation (Figs. 5*E*, and S6*F*), identifying Ser117 as the critical phosphorylation site. To definitively establish that CDK13 directly phosphorylates RBM39 at Ser117, an *in vitro* kinase assay was performed using purified recombinant proteins. As shown in Figure 5*F*, incubation of CDK13 with WT RBM39 in the presence of ATP resulted in a pronounced mobility shift indicative of phosphorylation, which was abrogated upon λ phosphatase treatment. In contrast, the S117A mutant exhibited no detectable phosphorylation under any condition. These results provide direct biochemical evidence that CDK13 specifically phosphorylates RBM39 at Ser117.

To determine if S117 phosphorylation is essential for CDK13-mediated resistance, we transfected cells with WT RBM39 or RBM39 S117A alongside CDK13 overexpression. Clonogenic assays showed that the S117A mutation significantly impaired the ability of CDK13 to confer cisplatin resistance compared to WT RBM39 (Fig. S6, *G* and *H*). IF staining for γH2AX confirmed that the S117A mutation reversed the suppression of cisplatin-induced DNA damage achieved by CDK13/WT RBM39 co-expression (Fig. S6, *I* and *J*). Alkaline comet assays yielded similar results (Fig. 5, *G* and *H*). Together, these data demonstrate that CDK13 enhances cisplatin resistance in EC cells by phosphorylating RBM39 at Ser117.

**Supplementary Figure 7:**
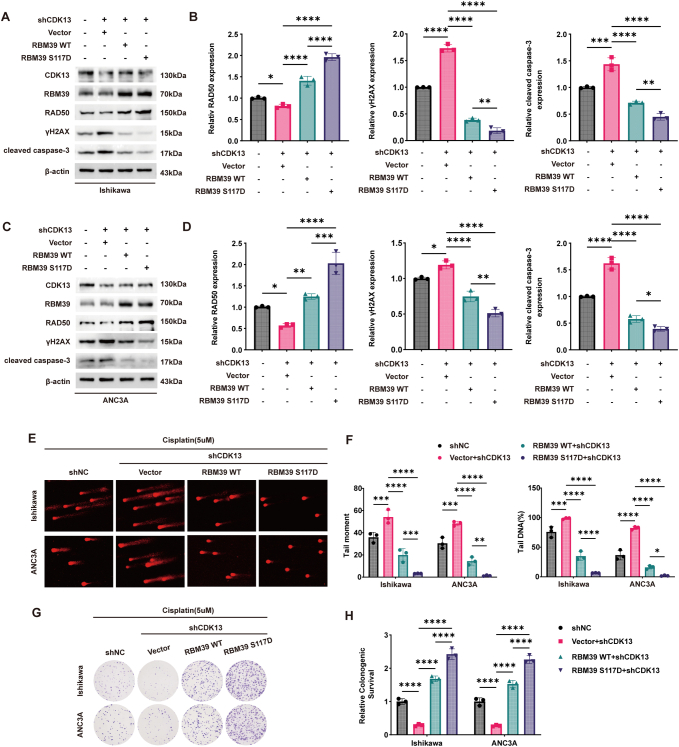


To assess whether Ser117 phosphorylation is sufficient for CDK13 function, we generated a phospho-mimetic mutant (RBM39 S117D) and expressed it in CDK13-knockdown cells. Western blot analysis showed that RBM39 S117D almost completely restored RAD50 expression and suppressed γH2AX and cleaved caspase-3 levels, outperforming WT RBM39 (Fig. S7, *A–D*). Consistently, RBM39 S117D significantly alleviated cisplatin-induced DNA damage in comet assays (Fig. S7, *E* and *F*) and enhanced clonogenic survival under cisplatin treatment (Fig. S7, *G* and *H*). These results demonstrate that the phospho-mimetic RBM39 S117D mutant rescues the defects caused by CDK13 knockdown and phenocopies CDK13 overexpression, confirming that Ser117 phosphorylation of RBM39 is a key functional determinant of the CDK13-RBM39-RAD50 axis.

### RBM39 stabilizes RAD50 mRNA in a phosphorylation-dependent manner and targeting this axis sensitizes tumors to cisplatin *in vivo*

**Figure 6:**
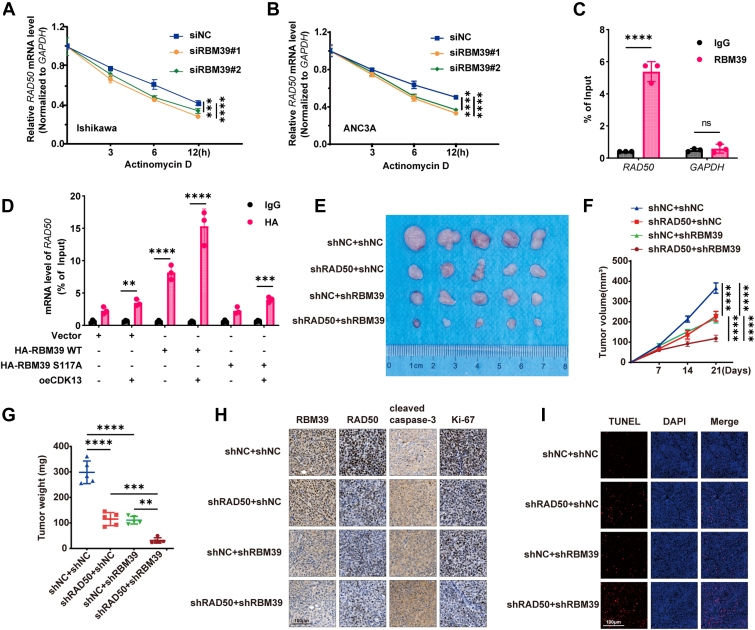
**RBM39 stabilizes RAD50 mRNA in a phosphorylation-dependent manner and targeting this axis sensitizes tumors to cisplatin *in vivo***. *A* and *B*, RAD50 mRNA stability was assessed by RT-qPCR in Ishikawa (*A*) and AN3CA (*B*) cells transfected with siNC or siRBM39 following treatment with actinomycin D (5 μg/ml). Data are presented as mean ± SD from three independent experiments, with individual data points superimposed. Statistical analysis was performed using two-way ANOVA with Tukey's *post hoc* test for multiple comparisons. ∗*p*< 0.05, ∗∗*p*< 0.01 *versus* siNC group. *C*, RNA immunoprecipitation assay using anti-RBM39 or control IgG antibody, followed by RT-qPCR analysis of enriched *RAD50* and *GAPDH* mRNAs. Data are presented as mean ± SD from three independent experiments, with individual data points superimposed. Statistical analysis was performed using one-way ANOVA with Tukey's *post hoc* test for multiple comparisons. ∗∗∗*p*< 0.001. *D*, RNA immunoprecipitation assay using HA antibody in Ishikawa cells transfected with the indicated constructs (Vector, HA-RBM39 WT, or HA-RBM39 S117A) with or without CDK13 overexpression. Enrichment of RAD50 mRNA was quantified by RT-qPCR and normalized to input. Data are presented as mean ± SD from three independent experiments, with individual data points superimposed. Statistical analysis was performed using one-way ANOVA with Tukey's *post hoc* test for multiple comparisons. ∗∗*p*< 0.01, ∗∗∗*p*< 0.001. *E* and *F*, xenograft tumor growth curves in nude mice injected with control (shNC), shRBM39, or shRAD50 EC cells. Data are presented as mean ± SEM (n = 8 per group). Tumor growth curves were analyzed using two-way repeated-measures ANOVA followed by Tukey's *post hoc* test. ∗*p*< 0.05, ∗∗*p*< 0.01, ∗∗∗*p*< 0.001 *versus* shNC group. *G*, final tumor weights at endpoint. Data are presented as mean ± SD, with individual data points superimposed. Statistical analysis was performed using one-way ANOVA with Tukey's *post hoc* test for multiple comparisons. ∗*p*< 0.05, ∗∗*p*< 0.01, ∗∗∗*p*< 0.001 *versus* shNC group. *H*, IHC staining for RBM39, RAD50, Ki-67, and cleaved caspase-3 in representative xenograft tumor tissues from each group. The scale bar represents = 100 μm. *I*, TUNEL staining (*green*) for apoptosis detection in representative xenograft tumor tissues from each group. Nuclei were counterstained with DAPI (*blue*). The scale bar represents = 100 μm. EC, endometrial cancer; IHC, immunohistochemical.

**Supplementary Figure 8:**
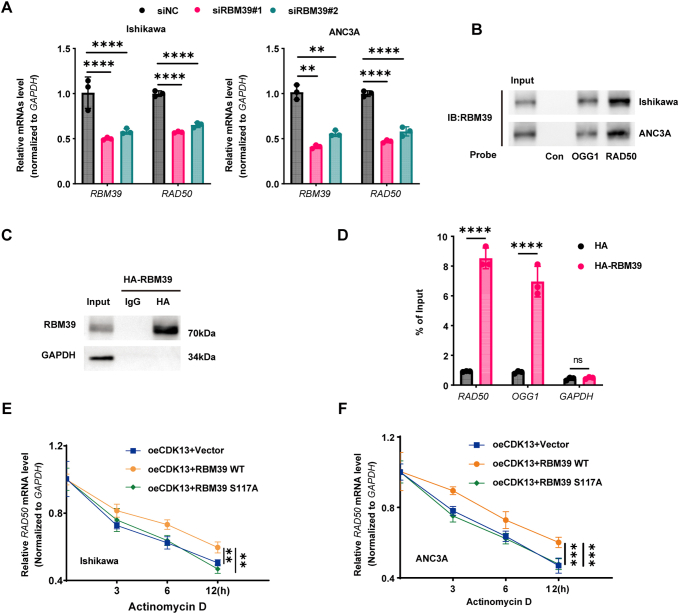


RBM39 is an RNA-binding protein known to stabilize target mRNAs by binding to their 3′-UTRs (24). We first confirmed that RBM39 knockdown significantly reduced *RAD50* mRNA levels (Fig. S8*A*). To assess *RAD50* mRNA stability, we inhibited new RNA synthesis with Actinomycin D and found that the depletion of RBM39 greatly reduced the half-life of *RAD50* mRNA (Fig. 6, *A* and *B*). RNA pull-down assays using probes specific for the RBM39 binding motif effectively retrieved RBM39 from Ishikawa and AN3CA cell lysates, using OGG1 as a positive control (20) (Fig. S8*B*). Conversely, RNA immunoprecipitation (RIP) assays using an RBM39 antibody demonstrated significant enrichment of *RAD50* mRNA, but not *GAPDH* mRNA (Fig. 6*C*). RIP assays performed in cells expressing HA-RBM39 confirmed the specific enrichment of *RAD50* and *OGG1* mRNAs by the HA antibody (Fig. S8, *C* and *D*). To investigate whether CDK13 enhances RBM39 binding to *RAD50* mRNA in a phosphorylation-dependent manner, we performed RIP assays using an HA antibody in cells coexpressing HA-RBM39 (WT or S117A) with or without CDK13. As shown in Figure 6*D*, CDK13 overexpression significantly increased the enrichment of *RAD50* mRNA bound to WT RBM39, but failed to enhance binding to the phosphorylation-deficient S117A mutant. These results demonstrate that CDK13 promotes RBM39-*RAD50* mRNA interaction specifically through Ser117 phosphorylation. Furthermore, the S117A mutation compromised the ability of RBM39 to stabilize *RAD50* mRNA, as evidenced by a faster decay rate in Actinomycin D chase experiments (Fig. S8, *E* and *F*). These findings indicate that RBM39 promotes RAD50 expression by stabilizing its mRNA, and phosphorylation at Ser117 is critical for this function.

To assess the therapeutic relevance of the CDK13-RBM39-RAD50 axis *in vivo*, we used Ishikawa cells with stable knockdown of RBM39, RAD50, or both to create xenograft models. Individual knockdown of either RBM39 or RAD50 significantly inhibited tumor growth. Notably, the dual knockdown of RBM39 and RAD50 resulted in the most profound suppression of tumor growth (Fig. 6, *E* and *F*). Tumor weight measurements corroborated these findings (Fig. 6*G*). IHC examination of xenograft tissues found that RBM39 knockdown diminished RAD50 and Ki-67 expression, while cleaved caspase-3 was elevated. Dual knockdown further enhanced these effects (Fig. 6*H*). TUNEL staining confirmed that individual knockdown of RBM39 or RAD50 increased apoptosis, and dual knockdown led to the highest level of TUNEL-positive cells (Fig. 6*I*). These results demonstrate that targeting the RBM39-RAD50 axis significantly inhibits EC tumor growth *in vivo*, with dual inhibition exhibiting a particularly potent effect.

In summary, our study delineates a novel signaling pathway in EC wherein upregulated CDK13 phosphorylates RBM39 at Ser117. This phosphorylation enhances the interaction between RBM39 and *RAD50* mRNA, leading to increased *RAD50* mRNA stability and protein expression. The consequent augmentation of DNA damage repair capacity promotes cisplatin resistance in tumor cells (Fig. S9). This identified axis presents potential therapeutic targets for overcoming platinum resistance in EC.

## Discussion

The development of cisplatin resistance represents a critical barrier to the successful treatment of advanced and recurrent EC, contributing significantly to its poor prognosis with 5-year survival rates as low as 17% (4, 29). While enhanced DNA damage repair capacity has been identified as a key mechanism underlying this resistance (8), the specific molecular drivers in EC remain incompletely understood. In this investigation, we delineate a novel pathway in which cyclin-dependent kinase 13 (CDK13), an emerging transcriptional regulator with oncogenic roles in other malignancies (14, 30), is a central player in conferring platinum resistance. We have shown that CDK13 is overexpressed in EC, and this heightened expression is related to resistance to platinum treatment. Mechanistically, we establish that CDK13 does not act alone but rather phosphorylates the RNA-binding protein RBM39 at Serine 117. This post-translational modification enhances the ability of RBM39 to bind and stabilize *RAD50* mRNA, a crucial gene in the DNA damage response. The resultant increase in RAD50 protein expression facilitates the repair of cisplatin-induced DNA lesions, thereby protecting tumor cells from apoptosis and diminishing the efficacy of chemotherapy. The identified CDK13/RBM39/RAD50 axis provides a mechanistic understanding of the clinical problem of cisplatin resistance and highlights potential new therapeutic targets to overcome this issue in endometrial carcinoma.

A primary mechanism underlying cisplatin resistance involves enhanced DDR capacity in tumor cells, which allows them to survive the cisplatin-induced DNA adducts and double-strand breaks (DSBs) (31). The MRE11–RAD50–NBS1 complex, a critical sensor of DSBs, initiates the DDR signaling cascade. Within this complex, RAD50 plays an indispensable role: it functions as an ATPase and a structural component that bridges DSB ends and activates the ATM kinase pathway, thereby orchestrating subsequent repair processes, predominantly homologous recombination (32). Recent investigations into gynecologic cancers underscore the clinical relevance of RAD50 and DNA repair pathways. For instance, uterine carcinosarcoma , a rare EC subtype, exhibits robust DNA repair capacity and elevated expression of DNA repair genes, a feature linked to its distinct therapeutic profile (33). Furthermore, in ovarian cancer, targeting DNA repair pathways—such as by shifting the balance from homologous recombination to the error-prone non-homologous end joining—has been shown to sensitize tumors to PARP inhibitors, highlighting the therapeutic vulnerability imposed by modulating DNA repair (34). In this context, our findings reveal a novel regulatory axis wherein CDK13 phosphorylates RBM39 at Ser117, enhancing its binding to *RAD50* mRNA and increasing RAD50 protein expression. By upregulating, the repair of DNA damage due to cisplatin is facilitated, as seen by reduced γH2AX foci and comet tail moments, thereby promoting tumor cell survival. It is noteworthy that our assessment of DNA damage relied on detecting γH2AX foci and DNA strand breaks *via* comet assays, both indicators of DSBs. While cisplatin primarily forms crosslinks, the cytotoxic efficacy of platinum-based chemotherapy is largely mediated through the conversion of unrepaired crosslinks into DSBs during DNA replication. These secondary DSBs are the direct triggers for the ATM/ATR kinase cascade and apoptotic signaling. Thus, our measurements captured the decisive, downstream lesions whose repair efficiency is ultimately governed by the CDK13/RBM39/RAD50 axis, directly linking the axis's function to cell fate determination upon cisplatin exposure.

Building upon this mechanistic understanding, critical questions and therapeutic opportunities emerge. While we have delineated how CDK13-mediated RBM39 phosphorylation enhances RAD50-dependent DNA repair, the upstream drivers of CDK13 overexpression in endometrial carcinoma remain elusive. Additionally, potential functional redundancy with other transcriptional CDKs implicated in DNA damage response, particularly CDK12, warrants further investigation to ascertain the unique role of CDK13 (30). Therapeutically, our findings position CDK13 inhibition as a compelling strategy to disrupt this resistance axis and restore cisplatin sensitivity, especially in tumors exhibiting high CDK13/RAD50 expression. This approach aligns with emerging paradigms of targeting DNA damage repair pathways to overcome chemoresistance in other malignancies (33). Future work should prioritize developing potent CDK13 inhibitors and evaluating their efficacy, both as single agents and in rational combinations with PARP inhibitors or other DNA-damaging agents. Ultimately, clinical validation of the CDK13/RBM39/RAD50 axis as a predictive biomarker could pave the way for personalized therapeutic strategies in advanced, platinum-resistant EC. While our study establishes a critical role for the CDK13-RBM39-RAD50 axis in mediating cisplatin resistance, we acknowledge that cisplatin is not the sole chemotherapeutic agent employed in the clinical management of EC. Whether this signaling axis also contributes to resistance against other first-line DNA-damaging therapeutics, such as carboplatin or doxorubicin, remains to be elucidated. Although cisplatin and carboplatin share a common mechanism of action involving the repair of DNA crosslinks by RAD50—suggesting the possibility of cross-resistance—the specific involvement of this axis in conferring resistance to agents with distinct mechanisms of action (*e*.*g*., topoisomerase II inhibitors) warrants further investigation.

In summary, this work defines the CDK13/RBM39/RAD50 axis as a key driver of platinum resistance in endometrial carcinoma. We demonstrate that CDK13 phosphorylates RBM39 at Ser117, enhancing its binding to *RAD50* mRNA and increasing RAD50 protein expression. This post-transcriptional regulation augments DNA damage repair capacity, enabling tumor cells to survive cisplatin-induced genotoxic stress. Our findings reveal a novel connection between kinase signaling and DNA repair pathways, identifying CDK13 and RBM39 as promising therapeutic targets. Targeting this axis, potentially through CDK13 inhibition or RBM39 degradation, represents a viable strategy to overcome platinum resistance in refractory EC.

## Experimental procedures

### Cell lines and cell culture

The human endometrial tumor cell lines Ishikawa (CL-0283, Procell) and ANC3A (CL-0505, Procell) were cultured in a humidified 37 °C, 5% CO_2_ incubator. Ishikawa cells were maintained in DMEM (Gibco, Cat# C11995500BT), while ANC3A cells were grown in MEM (Gibco, Cat# C12571500BT); both media were supplemented with 10% fetal bovine serum (Clark Bio), penicillin (100 U/ml), and streptomycin (100 μg/ml). All shRNA and siRNA constructs, along with the corresponding control oligonucleotides, were procured from GenePharma. Transfections were performed according to the manufacturer's instructions. The target sequences for shRNAs and siRNAs used in this study are listed in Tables S3 and S4, respectively.

### Patient samples

This study included tissue samples from 62 EC patients who underwent radical hysterectomy at the Department of Gynecology, the Affiliated Hospital of Qingdao University. All patients did not receive radiotherapy or chemotherapy before surgery. All diagnoses were verified by two independent pathologists, and the study received ethical approval from the hospital's Research Ethics Committee. (Permission No. QYFYWZLL30759), and Verbal consent was obtained from each patient.

### Xenograft tumor animal model

All animal experiments were approved by the Affiliated Hospital of Qingdao University, and every effort was undertaken to reduce animal suffering (14, 35). To establish stable knockdown cell lines, Ishikawa cells were infected with lentiviruses encoding shCDK13, shRBM39, shRAD50, or a combination thereof. Control cells underwent transfection with the matching empty vectors. For subcutaneous inoculation, a suspension of 1 × 10^6^cells in 0.1 ml of PBS was mixed with an equal volume of Matrigel (BD, 356234) on ice. The resulting 0.2 ml mixture (50% Matrigel) was injected into the lateral flanks of 6 to 8 weeks old female nude mice (Vitonlihua, 18–22*g*). Start a twice-weekly cisplatin (3 mg/kg, intraperitoneally [i.p.]) treatment for mice when the tumor is palpable. (36). The growth of the tumor was measured every 2 days with calipers, and its volume was calculated using the formula: (length × width^2^)/2. Upon euthanasia on day 21 or 28, tumor weight was measured, and the samples were collected for additional analysis, such as immunohistochemical staining and TUNEL assay. All animal studies were conducted in accordance with the Guide for the Care and Use of Laboratory Animals and approved by the Laboratory Animal Ethics Committee of the Affiliated Hospital of Qingdao University (Approval No. AHQU-MAL20250901YCX).

### Western blot analysis

As previously described (37), total protein extraction was performed with RIPA buffer containing a protease inhibitor mixture. The lysate supernatant was collected following centrifugation, and protein concentration was determined using a bicinchoninic acid protein assay kit (Thermo Fisher Scientific) according to the manufacturer's instructions. Equal amounts of protein (20–30 μg per lane) were loaded and separated by sodium dodecyl sulfate-polyacrylamide gel electrophoresis (SDS-PAGE). Following electrophoresis, proteins were transferred onto a polyvinylidene fluoride membrane (Millipore) using the WIX FastBlot system (Cat # 10112021). The membranes were blocked with 5% skim milk in Tris-buffered saline containing 0.1% Tween-20 for 2 h at room temperature, and subsequently incubated overnight at 4°C with primary antibodies against the following targets: CDK13 (1:500; 30461-1-AP, Proteintech), RBM39 (1:500; 21339-1-AP, Proteintech), RAD50 (1:2000; 29390-1-AP, Proteintech), γH2AX (1:5000; 83307-2-RR, Proteintech), and Cleaved Caspase-3 (1:500; 25128-1-AP, Proteintech). After washing with Tween-20, the membranes were incubated with horseradish peroxidase-conjugated secondary antibodies (1:10000; Rockland) for 1 h at room temperature. Immunoreactive bands were visualized using an enhanced chemiluminescence horseradish peroxidase substrate (Millipore), and chemiluminescent signals were captured using the Fusion Fx system (Vilber Lourmat). Densitometric analysis of the protein bands was performed using ImageJ software (https://imagej.net/ij/) (National Institutes of Health). The signal intensity of each target protein was normalized to that of β-actin or GAPDH, which served as the internal loading control. All experiments were repeated independently at least three times, and representative blots are shown. Quantitative data are presented as mean ± SD, and statistical analysis was carried out using one-way ANOVA followed by Tukey's *post hoc* test or Student's *t* test where appropriate, with a significance level set at *p* < 0.05.

### Quantitative real-time PCR

SevenFast Total RNA Extraction Kit for cells (Seven, #SM130) was used to extract total RNA. Using the Hieff first Strand cDNA Synthesis SuperMix for qPCR from YEASEN, total RNA was reverse-transcribed into first-strand cDNA following the removal of residual genomic DNA contaminants. A NanoDrop 2000 Spectrophotometer was used to quantify the quantity of RNA. Using a LightCycler96 system, qRT-PCR was carried out with diluted cDNA and Hieff qPCR SYBR Green Master Mix from YEASEN. For total RNA, GAPDH acted as the reference gene. The calculation of gene expression levels was done through the 2^−ΔΔ^Ct method (14, 38). The primer sequences for qRT-PCR are listed in Table S2.

### Apoptosis assay

After 48 h of exposure to cisplatin at a concentration of 5 μMcisplatin at a concentration of 5 μM, the cells were trypsinized and stained with Annexin V-FITC and propidium iodide (E-CK-A211, Elabscience) for 20 min at 4°C. Subsequently, flow cytometry analysis was performed to evaluate the ratio of apoptosis cells (Becton Dickinson) (39).

### Immunohistochemical staining and evaluation

Immunohistochemistry was conducted on 4-μm-thick paraffin-embedded tissue sections (40). Sections were treated with xylene for deparaffinization and rehydrated using a series of graded ethanol, followed by blocking with 10% normal goat serum. The samples were first treated with primary antibodies throughout the night at 4 °C. Subsequently, they were incubated with a rabbit IgG secondary antibody (021516, KPL) that was labeled with horseradish peroxidase. Chromogenic development was performed using a diaminobenzidine substrate kit. Then scan and analyze the stained sections.

### γ-H2AX foci formation assay

Although cisplatin primarily induces DNA crosslinks, its cytotoxicity is largely mediated through the subsequent formation of DSBs during replication. The formation of γH2AX foci is a well-established marker for these DSBs, reflecting the downstream lethal DNA damage. Cells transfected with shCDK13 or control shRNA were exposed to cisplatin (5μM) for 48 hsplatin (5 μM) *for 48 hours* to measure DNA damage as previous described (41). Cells were fixed in 4% paraformaldehyde for 20 min after treatment and permeabilized with 0.5% Triton X-100 in PBS for 15 min. After that, the cells were exposed to the primary γ-H2AX antibody (Proteintech, Cat# 83307-2-RR) overnight at 4 °C. Following PBS washing, the cells were exposed to an Alexa Fluor 488 conjugated secondary antibody in the dark at room temperature for an hour. DAPI (SouthernBiotech, Cat No.0100–20) was used to counterstain nuclei for five minutes. Using a fluorescent microscope, foci development was seen.

### Cell proliferation assay and determination of half maximal inhibitory concentration (IC50)

For the proliferation assay, 3000 cells were seeded into a 96 well plate and survival rates were evaluated for 3 consecutive days using Cell Counting Kit-8 (SC-119, SEVEN) according to the manufacturer's guidelines (at least three replicates per treatment group) (41). Each well received 100 μl of CCK8 reagent and culture media made in a 1:9 ratio. The combination was then incubated in the incubator for 0.5 to 3 h. For cytotoxic assays, cells were cultured for 24 h before treatment with varying concentrations of cisplatin for 48 h. Measurements of absorbance were taken at 450 nm, and IC50 values were calculated using GraphPad Prism 9.5 (GraphPad Software, https://www.graphpad.com).

### Colony formation assays

In 6-well plates, 1000 cells were plated and cultured for 14 days at 37 °C in a 5% CO2 atmosphere as previous described (35, 42). The cells were then washed with PBS, fixed in 4% paraformaldehyde for 15 mins, and stained with crystal violet for 30 min. ImageJ was used to count any cluster with over 50 cells as one colony.

### 5-Ethynyl-2′-deoxyuridine (EdU) assay

According to the manufacturer's guidelines, cell proliferation was assessed using the EdU Proliferation Kit (C0075S, Beyotime) (43). Transfected EC cells were briefly seeded in 96-well plates and cultured for 12 h. After a 2-h exposure to 50 μM EdU, the cells were fixed using 4% paraformaldehyde and stained with Apollo Dye Solution and Hoechst 33342 for nuclear counterstaining. Fluorescent images were captured and quantitatively assessed using a ZEISS Axioscope5 microscope (ZEISS Microscopy, https://www.zeiss.com/microscopy), with each experiment conducted three times.

### Application of cisplatin

Cisplatin was purchased from Solarbio. Cisplatin was dissolved in DMF (HY-Y0345, MCE) to prepare a stock solution (1 mM). Following the manufacturer's guidelines, a working solution was prepared and utilized to assess its impact on the proliferation of EC cells (44).

### Alkaline comet assays

The alkaline comet assay was used to assess DNA damage at the level of individual cells. Cells were treated with cisplatin (5 μM) for 48 h prior to trypsinization. After trypsinization, the cells were resuspended to a concentration of 1 × 10^6^/ml. Further steps were conducted using a commercial DNA damage comet assay kit (Beyotime, C2041M) in line with the manufacturer's instructions (45). Briefly, cells were trapped in 0.7% low melting point agarose and applied to slides that were already coated with 1% normal melting point agarose. After lysis and alkaline unwinding, electrophoresis was performed at 25 V for 30 min. Then, the DNA was stained with propidium iodide. Fluorescence images were acquired with an Olympus IX74 microscope, and parameters including tail moment and tail DNA (%) were quantified using CASP software (46).

### Co-immunoprecipitation assay

Co-immunoprecipitation (Co-IP) experiments were conducted with the Pierce Classic Magnetic IP/Co-IP Kit (Thermo Fisher Scientific, #88804) or Anti-Flag/HA Magnetic Beads (Beyotime, P2115, P2121) as previous described (35, 42). Following lysis of cultured cells on ice, the lysates were subjected to a washing buffer treatment. Specific antibodies, including HA, were used to incubate the lysates for one hour at room temperature, followed by the addition of pre-cleaned magnetic beads and another hour of incubation. The complexes of antibodies and proteins were isolated with a magnetic rack, cleaned, and eluted using an elution buffer. Western blot or mass spectrometry was used to analyze the bound proteins subsequently to identify their interacting partners.

### RIP assay

Cell lysates were subjected to immunoprecipitation after being lysed in RIP buffer with protease and RNase inhibitors (14, 35). Incubation with specific antibodies, including RBM39 and HA, or an isotype control was performed at 4 °C for four hours, followed by the addition of Protein A/G magnetic beads for two more hours. Following the washing of the complexes with RIP buffer, TRIzol reagent was used to extract the co-precipitated RNA. After isolation, the RNA was analyzed using RT-qPCR or RNA sequencing to detect transcripts bound to proteins.

### RNA stability assay

To evaluate RNA stability, transcription was inhibited with 5 μg/ml actinomycin D (HY-17559, MCE). Cells, whether RBM39 was modulated or not, were harvested at set times (0, 4, 8, 12 h) after treatment. The total RNA was extracted and analyzed using qRT-PCR to assess RNA decay kinetics (47).

### Phosphoproteomic analysis by LC-MS/MS

Phosphoproteomic analysis was performed to identify downstream phosphorylation targets of CDK13. Briefly, Ishikawa cells stably expressing control shRNA or shRNA targeting CDK13 were lysed in a urea-based lysis buffer (8 M urea, 50 mM Tris-HCl pH 8.0) supplemented with protease and phosphatase inhibitors. The protein concentration was determined using a bicinchoninic acid assay. Subsequently, proteins were reduced, alkylated, and digested with trypsin overnight. The resulting peptides were desalted and subjected to phosphopeptide enrichment using TiO2 beads, according to established protocols (48). Enriched phosphopeptides were analyzed by nanoflow LC-MS/MS on a Q Exactive HF mass spectrometer (Thermo Fisher Scientific) coupled to an EASY-nLC 1200 system. Peptides were separated on a reversed-phase column (75 μm × 25 cm, Reprosil-Pur C18-AQ, 1.9 μm) with a 120-min gradient from 5 to 35% acetonitrile in 0.1% formic acid at a flow rate of 300 nl/min. Raw data were processed using MaxQuant software (version 1.6.17.0) with the Andromeda search engine (MaxQuant, https://www.maxquant.org). MS/MS spectra were searched against the human UniProt database (Swiss-Prot, reviewed). Search parameters included: precursor mass tolerance of 10 ppm, fragment mass tolerance of 0.02 Da; trypsin specificity with up to two missed cleavages; carbamidomethylation of cysteine was set as a fixed modification; protein N-terminal acetylation and oxidation of methionine were set as variable modifications. False discovery rate was set to <1% at both peptide and protein levels. For phosphorylation site localization, the post-translational modification scoring algorithm integrated in MaxQuant was applied. Sites with post-translational modification localization probability ≥ 0.75 were considered confidently localized and retained for downstream analysis. Quantification was performed using the label-free quantification algorithm. For each phosphopeptide, raw intensities were log_2_-transformed, and fold-changes were calculated as the ratio of mean intensities between shCDK13 and shNC groups. Statistical significance was assessed by unpaired two-tailed Student's *t* test. Phosphopeptides with |log_2_FC| ≥ 0.26 (equivalent to fold change ≥ 1.2) and *p*< 0.05 were considered significantly regulated by CDK13 knockdown.

### *In vitro* kinase assay

The MBP-tagged CDK13 and its binding partner cyclin K were coexpressed in *E*. *coli* BL21 (DE3) and purified using amylose resin (New England Biolabs, E8022S) according to the manufacturer's instructions. HA-tagged WT RBM39 (RBM39-WT) and the phosphorylation-defective S117A mutant (RBM39-S117A) were expressed in HEK293T cells and purified by immunoprecipitation using anti-HA affinity agarose (Thermo Fisher Scientific, #26181), followed by elution with HA peptide.

For the kinase reaction, purified MBP-CDK13/cyclin K complex (200 ng) was incubated with purified HA-RBM39 (WT or S117A, 500 ng) in 30 μl of kinase buffer (50 mM Hepes pH 7.5, 10 mM MgCl_2_, 2 mM DTT, 1 mM Na_3_VO_4_) supplemented with 100 μM ATP. The reaction was carried out at 30 °C for 60 min. To establish negative controls, the following treatments were performed (1): omission of ATP from the reaction mixture to demonstrate the dependence of the phosphorylation signal on kinase activity; and (2) addition of λ protein phosphatase (λPP, 400 U, New England Biolabs, P0753S) after the kinase reaction, followed by a further 30-min incubation at 30 °C to confirm that the observed phosphorylation signal could be reversed by a specific phosphatase. All reactions were terminated by adding SDS loading buffer and boiling for 5 min. The phosphorylation status of RBM39 was assessed by Phos-tag SDS-PAGE. Samples were separated on an 8% SDS-polyacrylamide gel containing 50 μM Phos-tag reagent and 100 μM MnCl_2_ (Vazyme, Phos-assay Acrylamide). After electrophoresis, the gel was washed with transfer buffer containing 1 mM EDTA and subjected to Western blot analysis using an anti-HA antibody (Proteintech, 1:1000) to detect RBM39 and its phosphorylated forms. Parallel samples were analyzed by conventional SDS-PAGE, followed by immunoblotting with anti-MBP (Cell Signaling Technology, #2396S, 1:1000) and anti-HA antibodies to confirm the presence of CDK13 and consistent loading of RBM39 protein, respectively.

### Statistical analysis

All experiments were performed with at least three independent biological replicates. Quantitative data are presented as mean ± SD. Statistical analyses and graphing were conducted using GraphPad Prism 9.5 and IBM SPSS Statistics 26.0.

Prior to analysis, normality (Shapiro–Wilk test) and homogeneity of variances (Levene’s test) were assessed. Outlier analysis was performed using the ROUT method (Q = 1%); no outliers were identified in any dataset, and therefore all data points were included in the final analysis. For comparisons between two groups, the unpaired two-tailed Student’s ∗t∗-test was used when data met parametric assumptions; otherwise, the non-parametric Mann–Whitney U test was applied. For comparisons among three or more groups, one-way ANOVA was performed when parametric assumptions were satisfied, followed by Tukey’s *post hoc* test for multiple comparisons. If parametric assumptions were violated, the Kruskal–Wallis H test was used, followed by Dunn’s *post hoc* test for multiple comparisons. For experiments involving two independent variables, two-way ANOVA was employed, followed by Tukey’s *post hoc* test for multiple comparisons. Repeated-measures ANOVA was applied for longitudinal data, with Tukey’s *post hoc* test for multiple comparisons where appropriate. The association between CDK13 expression and clinicopathological parameters was analyzed using Pearson’s chi-square test or Fisher’s exact test. Statistical significance was set at *p*< 0.05, and significance levels in figures are denoted as follows: *p*< 0.05, ∗*p*< 0.01, ∗∗*p*< 0.001, ∗∗∗*p*< 0.0001.

## Ethics statement

All methods in this study were performed in accordance with the relevant guidelines and regulations. The study involving human participants was reviewed and approved by the Ethics Committee of the Affiliated Hospital of Qingdao University (Permission No. QYFYWZLL30759). All patients provided written informed consent prior to participation. All animal experimental procedures were approved by the Committee for the Care and Use of Laboratory Animals at the Affiliated Hospital of Qingdao University (Approval No. AHQU-MAL20250901YCX) and were carried out in compliance with the Guide for the Care and Use of Laboratory Animals (National Research Council).

## Data availability

The datasets used for Figure S1, *A* and *B* were analyzed using the UALCAN platform (49). All other data supporting the findings of this study are available in the main figures or supporting information.

## Supporting information

This article contains supporting information.

## Conflict of interest

The authors declare that they have no conflicts of interest with the contents of this article.

## Figures and Tables

**Supplementary Figure 9:**