# Supplementary material for: Phosphorylation of RBM39 by CDK13 stabilizes RAD50 mRNA to drive cisplatin resistance in endometrial cancer
Source: J Biol Chem. 2026 Apr 15;302(6):111447. doi: 10.1016/j.jbc.2026.111447 (PMC13196387; doi:10.1016/j.jbc.2026.111447)
Supplement: Supplementary table 1 [file mmc1.docx]

**Supplementary table 1**. Clinical Characteristics and CDK13 Expression

|  |  | CDK13 expression | |  |
| --- | --- | --- | --- | --- |
| Characteristics | Number of patients (n) | Low (%) | High (%) | P value |
| **No. of patients** | 62 | 31 | 31 |  |
| **Age** |  |  |  |  |
| ≤60 | 30 | 16 (51.6) | 14 (45.2) | 0.611 |
| >60 | 32 | 15 (48.4) | 17 (54.8) |  |
| **pT status** |  |  |  |  |
| pT1a | 41 | 26 (83.9) | 15 (48.4) | 0.003 |
| ≥pT1b | 21 | 5 (16.1) | 16 (51.6) |  |
| **pN status** |  |  |  |  |
| pN0 | 47 | 25 (80.6) | 22 (71.0) | 0.374 |
| pN1 | 15 | 6 (19.4) | 9 (29.0) |  |
| **pM stage** |  |  |  |  |
| pM0 | 43 | 24(77.4) | 19(61.3) | 0.168 |
| pM1 | 19 | 7(22.6) | 12(38.7) |  |
| **Grade** |  |  |  |  |
| G1-G2 | 48 | 26 (83.9) | 22 (71.0) | 0.224 |
| G3-G4 | 14 | 5 (16.1) | 9 (29.0) |  |
