# Supplementary material for: Phosphorylation of RBM39 by CDK13 stabilizes RAD50 mRNA to drive cisplatin resistance in endometrial cancer
Source: J Biol Chem. 2026 Apr 15;302(6):111447. doi: 10.1016/j.jbc.2026.111447 (PMC13196387; doi:10.1016/j.jbc.2026.111447)
Supplement: Supplementary table 2 [file mmc2.docx]

***Supplementary table 2*.** Oligos used in the study

| Gene | forward | reverse |
| --- | --- | --- |
| CDK13 | 5’-CCCCTAGTCCCTACAGCAG-3’ | 5’-GCCTAGATGAATACGGGCTTCTG-3’ |
| RBM39 | 5’-TGGGAACAACTGGTCGTCTT-3’ | 5’-ATAACAAAAGAGAATTCTGCCACA-3’ |
| RAD50 | 5’-CAGTCATGTTGCCCCGTTTG-3’ | 5’-TCTGGAGCAAGTCGCAGTTT-3’ |
| XRCC3 | 5’-GGAAGAGGAGTGCGGAACC-3’ | 5’-GGCTGACTTGACTGAGGCAT-3’ |
| GAPDH | 5’-GCACCGTCAAGGCTGAGAAC-3’ | 5’-TGGTGAAGACGCCAGTGGA-3’ |
| MSH3 | 5’-TCTGGGAATGTCTGGCAACT-3’ | 5’-AGGAAGGGCAGAATCGCAG-3’ |
| MUTYH | 5’-GCCTCTAGAACTATGAGCCCG-3’ | 5’-CCTCATGATGGCCCACAGAC-3’ |
| FANCI | 5’-TCAAACCCTGAGAGAAGGTGA-3’ | 5’-AATCCCCCGATTCCACCAAC-3’ |
| FANCL | 5’-TCAAGTTGAAGGCAAAGTATCCTG-3’ | 5’-TTTACCTGAGGTGTCCAGGAGG-3’ |
| RAD51D | 5’-ACAGTGGTGGACCTGGTTTC-3’ | 5’-GAGCATTCCTGACCCCACTC-3’ |
| MCM8 | 5’-GACCTCTGTCCCAAGCAAGT-3’ | 5’-TCATCTCTCCTGTGCTTCTTC-3’ |
| MCM9 | 5’-CGCCTCACCAGCCTTCTTG-3’ | 5’-TCTAGGTAATGGCTGTGTCAGA-3’ |
| ATR | 5’-CCAAAGCGCCACTGAATGAA-3’ | 5’-ACGGCAGTCCTGTCACTCTA-3’ |
| FIRRM | 5’-TTGGCGGGTCTGGTTTGAAG-3’ | 5’-AAGCCAACAACAAGCGGAAG-3’ |
| FAN1 | 5’-TGGCCCCAGGAAGAAGAAAT-3’ | 5’-GACTTGAAATCACCAAAAGTGGGA-3’ |
| DCLRE1C | 5’-AGACCGCTTCGATAGGGAGA-3’ | 5’-CAAGCTGCACTCCAACCTTC-3’ |
| SERBP1 | 5’-CCTGGGCACTTACAGGAAGG-3’ | 5’-GGTCCGATTCGTCGTCAAATAAC-3’ |
| TMEM87B | 5’-CTGCATCAGATTGGCCCCTAA-3’ | 5’-TCGCAGAAATCAACTCCGCAA-3’ |
| USP7 | 5’-GGAAGCGGGAGATACAGATGA-3’ | 5’-AAGGACCGACTCACTCAGTCT -3’ |
| FCHO2 | 5’-ATGGTCATGGCGTATTTCGTC-3’ | 5’-TGGTAGCTCGTTCCCTTACAAA-3’ |
| CDC6 | 5’-CCAGGCACAGGCTACAATCAG-3’ | 5’-AACAGGTTACGGTTTGGACATT-3’ |
| RFX5 | 5’-GATGAGCCTGATGCTAAGAGC-3’ | 5’-GGGAGCTGAAGGTAGAGATACA-3’ |
| PLEC | 5’-CGATGCGACAACTTCACCTC-3’ | 5’-GCCGGTACACCTTGTTCATGT-3’ |
| TEX264 | 5’-ATGTCGGACCTGCTACTACTG-3’ | 5’-GCCCCATGTGGAACTTGTAGG-3’ |
| HRH1 | 5’-AGATGTGTGAGGGCAACAAGA-3’ | 5’-CAAGCAGATAGTGCTCAGGAC-3’ |
