# Supplementary material for: Phosphorylation of RBM39 by CDK13 stabilizes RAD50 mRNA to drive cisplatin resistance in endometrial cancer
Source: J Biol Chem. 2026 Apr 15;302(6):111447. doi: 10.1016/j.jbc.2026.111447 (PMC13196387; doi:10.1016/j.jbc.2026.111447)
Supplement: Supplementary table 3 [file mmc3.docx]

***Supplementary table 3*.** Sequences of siRNAs used in this study.

| Gene | sense（5'-3'） | antisense（5'-3'） |
| --- | --- | --- |
| siRBM39#1 | CUGGUCGUCUUCAGUUAAUTT | AUUAACUGAAGACGACCAGTT |
| siRBM39#2 | GAGAGAACCUAUUGAUAAUTT | AUUAUCAAUAGGUUCUCUCTT |
| siRAD50#1 | GCGACUUGCUCCAGAUAAATT | UUUAUCUGGAGCAAGUCGCTT |
| siRAD50#2 | GGGAUUCAAUGUUCAUUAATT | UUAAUGAACAUUGAAUCCCTT |
| siSERBP1#1 | GGACUUGUGUGGAGAAUUGTT | CAAUUCUCCACACAAGUCCTT |
| siSERBP1#1 | CCAGAGAUUAAUUUAGUCCTT | GGACUAAAUUAAUCUCUGGTT |
| siTMEM87B#1 | GCCCTTAATAGATGATTCTGATT | CAGAATCATCTATTAAGGGCATT |
| siTMEM87B#2 | CACAAACTATGAAGACCCTAATT | TAGGGTCTTCATAGTTTGTGTT |
| siUSP7#1 | CGUGGUGUCAAGGUGUACUTT | AGUACACCUUGACACCACGTT |
| siUSP7#2 | UGUAUCUAUUGACUGCCCUTT | AGGGCAGUCAAUAGAUACATT |
| siFCHO2#1 | GGAAGAACAAGUAAAGUCUTT | AGACUUUACUUGUUCUUCCTT |
| siFCHO2#2 | GAAAUCCAAGGAAAAUUACTT | GUAAUUUUCCUUGGAUUUCTT |
| siCDC6#1 | CCAAACUGGAGCUGAAAUATT | UAUUUCAGCUCCAGUUUGGTT |
| siCDC6#2 | GCAUCGUCAAAGAUUAUUATT | UAAUAAUCUUUGACGAUGCTT |
| siRFX5#1 | GGAAGCCAAAAGGAGGCUUUTT | AAAGCCUCCUUUUGGCUUCCTT |
| siRFX5#2 | GGAAGAGUGUUUAUAUCCCTT | GGGGAUAUAAACACUCUUCCTT |
| siPLEC#1 | CCUCAACUGUAUUUCCUACGTT | CGUAGGAAAUACAGUUGUGATT |
| siPLEC#2 | CCACAGACUCUGUCAUCUATT | UAGAUGACAGAGUCUGUGG TT |
| siTEX264#1 | GGAAGCUUCAGUAUCUCAATT | UUGAGAUACUGAAGCUUCCTT |
| siTEX264#2 | CCUGAAGCUCAGAUCCAAATT | UUUGGAUCUGAGCUUCAGGTT |
| siHRH1#1 | GAGAUAUCAGAGGAUCAGATT | UCUGAUCCUCUGAUAUCUCTT |
| siHRH1#2 | GCAAAUUGAGGAGUGGGUCTT | GACCCACUCCUCAAUUUGCTT |
| siPOLR2A#1 | GAGCUGUACCACGUCAUCUTT | AGAUGACGUGGUACAGCUCTT |
| siPOLR2A#2 | GCACGUUAAUGAGGACUCUTT | AGAGUCCUCAUUAACGUGCTT |
| siCDK12#1 | GCCCAAUUCAGAGAGACAUTT | AUGUCUCUCUGAAUUGGGCTT |
| siCDK12#1 | GGGAACAAGAGACUCUAAATT | UUUAGAGUCUCUUGUUCCCTT |
