# Supplementary material for: Phosphorylation of RBM39 by CDK13 stabilizes RAD50 mRNA to drive cisplatin resistance in endometrial cancer
Source: J Biol Chem. 2026 Apr 15;302(6):111447. doi: 10.1016/j.jbc.2026.111447 (PMC13196387; doi:10.1016/j.jbc.2026.111447)
Supplement: Supplementary table 4 [file mmc4.docx]

***Supplementary table 4*.** Sequences of shRNAs.

| Gene | Target sequence (5'→3') |
| --- | --- |
| shCDK13#1 | GCTGATAGCTTACGAGGAAAT |
| shCDK13#2 | CGATGTCTTCTTGCTGATTTA |
| shCDK13#3 | GCTGCGCTAGACTTATTTGAT |
| shRBM39#1 | GCTTCGAGTGCTAGTTCATTT |
| shRBM39#2 | GCGAAGTAGAGACAGAGAAAG |
| shRBM39#3 | GGATGATGTGATTGAAGAATG |
| shRAD50#1 | GCTGTGCTAAATAATGTCATT |
| shRBM39#2 | GCCAAATTGAACAAGGAACTA |
| shRBM39#3 | CGCCTAAAGAACGACATAGAA |
