# Supplementary material for: Phosphorylation of RBM39 by CDK13 stabilizes RAD50 mRNA to drive cisplatin resistance in endometrial cancer
Source: J Biol Chem. 2026 Apr 15;302(6):111447. doi: 10.1016/j.jbc.2026.111447 (PMC13196387; doi:10.1016/j.jbc.2026.111447)
Supplement: Supplementary Figure Legends [file mmc5.docx]

**Supplemental Figure Legends**

**Fig. 1:CDK13 protein expression in EC from public databases (related to Figure 1).**

1. Densitometric quantification of CDK13 protein levels from (Figure 1B). Band intensities were quantified using ImageJ software and normalized to β-actin. Data are presented as mean ± SD from three independent experiments, with individual data points superimposed. Statistical analysis was performed using unpaired two-tailed Student's t-test. *p < 0.05, **p < 0.01, ***p < 0.001, ****p < 0.0001. (B) Analysis of CDK13 protein expression in UCEC using the UALCAN platform. Statistical significance was determined by the UALCAN platform using unpaired two-tailed Student’s t-test. ***P < 0.001. (C) CDK13 protein expression levels across different histological grades of UCEC from the UALCAN platform (Grade 1, n = 35; Grade 2, n = 54; Grade 3, n = 76). Statistical significance between any two groups was determined by the UALCAN platform using unpaired two-tailed Student’s t-test. *P < 0.05, **P < 0.01, ***P < 0.001, ****P < 0.0001.

**Fig. 2:Validation of CDK13 manipulation and functional assays in EC cells (related to Figure 2).**

(A, B) RT-qPCR analysis confirming CDK13 knockdown (A) and overexpression (B) in Ishikawa and AN3CA cells. Data are presented as mean ± SD from three independent experiments, with individual data points superimposed. Statistical analysis was performed using unpaired two-tailed Student's t-test. *p < 0.05, **p < 0.01, ***p < 0.001, ****p < 0.0001 vs. corresponding control. (C, D) Western blot analysis confirming CDK13 knockdown (C) and overexpression (D) in Ishikawa and AN3CA cells. (E, F) Densitometric quantification of CDK13 protein levels from (C). Band intensities were quantified using ImageJ software and normalized to β-actin. Data are presented as mean ± SD from three independent experiments, with individual data points superimposed. Statistical analysis was performed using unpaired two-tailed Student's t-test. *p < 0.05, **p < 0.01, ***p < 0.001, ****p < 0.0001. (G, H) Densitometric quantification of CDK13 protein levels from (D). Band intensities were quantified using ImageJ software and normalized to β-actin. Data are presented as mean ± SD from three independent experiments, with individual data points superimposed. Statistical analysis was performed using unpaired two-tailed Student's t-test. *p < 0.05, **p < 0.01, ***p < 0.001, ****p < 0.0001. (I, J) Quantification of EdU-positive cells in CDK13-knockdown (I) and CDK13-overexpressing (J) EC cells. Data are presented as mean ± SD from three independent experiments, with individual data points superimposed. Statistical analysis was performed using unpaired two-tailed Student's t-test. *p < 0.05, **p < 0.01, ***p < 0.001, ****p < 0.0001 vs. corresponding control. (K) Cell proliferation was assessed by CCK-8 assay in CDK13-overexpressing EC cells. Data are presented as mean ± SD from three independent experiments, with individual data points superimposed. Statistical analysis was performed using two-way ANOVA with Tukey's post-hoc test for multiple comparisons. *p < 0.05, **p < 0.01, ***p < 0.001 vs. corresponding control. (L-O) Clonogenic formation assays in CDK13-knockdown (L, M) and CDK13-overexpressing (N, O) EC cells treated with or without cisplatin. (L, N) Representative images of colony formation. (M, O) Quantification of colony formation assays. Data are presented as mean ± SD from three independent experiments, with individual data points superimposed. Statistical analysis was performed using one-way ANOVA with Tukey's post-hoc test for multiple comparisons. *p < 0.05, **p < 0.01, ***p < 0.001, ****p < 0.0001.

**Fig. 3:CDK13 regulates apoptosis and DNA damage in EC cells (related to Figure 3).**

(A, B) Apoptosis was analyzed by flow cytometry in CDK13-manipulated ANC3A cells treated with or without cisplatin (5 µM) for 48 hours. Representative plots (A) and quantification (B) are shown. Data are presented as mean ± SD from three independent experiments, with individual data points superimposed. Statistical analysis was performed using one-way ANOVA with Tukey's post-hoc test for multiple comparisons. *p < 0.05, **p < 0.01, ***p < 0.001. (C) Quantification of γH2AX foci (green) in EC cells treated with cisplatin (5 µM) for 24 hours. Nuclei were stained with DAPI (blue). Scale bar = 20 µm. Data are presented as mean ± SD from three independent experiments, with individual data points superimposed. Statistical analysis was performed using one-way ANOVA with Tukey's post-hoc test for multiple comparisons. *p < 0.05, **p < 0.01, ***p < 0.001, ****p < 0.0001. (D-K) Quantification of γH2AX (D-F) and cleaved caspase-3 (G-I) protein levels in CDK13-manipulated EC cells. Ishikawa and AN3CA cells were transfected with shNC, shCDK13, vector, or oeCDK13 and treated with cisplatin (5 µM) for 48 h. Representative Western blots are shown in Figure 3E. Band intensities were quantified using ImageJ software and normalized to β-actin as a loading control. Data are presented as mean ± SD from three independent experiments, with individual data points superimposed. Statistical analysis was performed using one-way ANOVA with Tukey's post-hoc test for multiple comparisons. *p < 0.05, **p < 0.01, ***p < 0.001, ****p < 0.0001.

**Fig. 4:RAD50 mediates CDK13-induced cisplatin resistance in EC cells(related to Figure 4).**

1. Relative mRNA expression of indicated DNA damage repair-related genes in Ishikawa and AN3CA cells transfected with oeCDK13 or control vector, measured by RT-qPCR. (B) Quantification of colony formation assays in EC cells co-transfected with oeCDK13 and/or siRAD50. Data are presented as mean ± SD from three independent experiments, with individual data points superimposed. Statistical analysis was performed using one-way ANOVA with Tukey's post-hoc test for multiple comparisons *p < 0.05, **p < 0.01, ***p < 0.001, ****p < 0.0001. (C, D) Flow cytometry analysis of apoptosis in the indicated EC cells treated with or without cisplatin (5 µM) for 48 hours. Data are presented as mean ± SD from three independent experiments, with individual data points superimposed. Statistical analysis was performed using one-way ANOVA with Tukey's post-hoc test for multiple comparisons. *p < 0.05, **p < 0.01, ***p < 0.001, ****p < 0.0001. (E) Quantification of immunofluorescence images of γH2AX foci (green) in the indicated EC cells treated with cisplatin (5 µM) for 24 hours. Nuclei were stained with DAPI (blue). Scale bar = 20 µm. Data are presented as mean ± SD from three independent experiments, with individual data points superimposed. Statistical analysis was performed using one-way ANOVA with Tukey's post-hoc test for multiple comparisons. *p < 0.05, **p < 0.01, ***p < 0.001, ****p < 0.0001. (F-I) Densitometric quantification of cleaved caspase-3(F, G) and γH2AX(H, I) protein levels from Figure 4F. Band intensities were quantified using ImageJ software and normalized to β-actin as a loading control. Data are presented as mean ± SD from three independent experiments, with individual data points superimposed. Statistical analysis was performed using one-way ANOVA with Tukey's post-hoc test for multiple comparisons. *p < 0.05, **p < 0.01, ***p < 0.001, ****p < 0.0001. (J) Quantification of images of the alkaline comet assay performed in the indicated EC cells treated with cisplatin (5 µM) for 48 hours. Scale bar = 20 µm. Data are presented as mean ± SD from three independent experiments, with individual data points superimposed. Statistical analysis was performed using one-way ANOVA with Tukey's post-hoc test for multiple comparisons. *p < 0.05, **p < 0.01, ***p < 0.001, ****p < 0.0001.

**Fig. 5:RBM39 is a critical downstream target of CDK13 and its phosphorylation at Ser117 is required for cisplatin resistance (related to Figure 5).**

1. *RAD50* mRNA levels measured by RT-qPCR in Ishikawa and AN3CA cells co-transfected with oeCDK13 and siRNAs targeting candidate factors. Data are presented as mean ± SD from three independent experiments, with individual data points superimposed. Statistical analysis was performed using one-way ANOVA with Tukey's post-hoc test for multiple comparisons. *p < 0.05, **p < 0.01, ***p < 0.001, ****p < 0.0001 vs. oeCDK13 + siNC group. (B) Correlation analysis between *RBM39* and *RAD50* mRNA expression in EC tissues from the TCGA dataset (n = 543). Pearson's correlation coefficient and P-value are shown. (C, D) Colony formation assays were performed in EC cells co-transfected with oeCDK13 and/or siRBM39 and treated with cisplatin. (C) Representative images of colony formation. (D) Quantification of colony formation. Data are presented as mean ± SD from three independent experiments, with individual data points superimposed. Statistical analysis was performed using one-way ANOVA with Tukey's post-hoc test for multiple comparisons. *p < 0.05, **p < 0.01, ***p < 0.001, ****p < 0.0001. (E, F) IC50 values for cisplatin determined by CCK-8 assay in the indicated Ishikawa (E) and AN3CA (F) cells. Data are presented as mean ± SD from three independent experiments, with individual data points superimposed. Statistical analysis was performed using one-way ANOVA with Tukey's post-hoc test for multiple comparisons. *p < 0.05, **p < 0.01, ***p < 0.001. (G, H) Flow cytometry analysis of apoptosis in the indicated EC cells treated with cisplatin (5 µM) for 48 hours. (G) Representative flow cytometry plots. (H) Quantification of apoptosis. Data are presented as mean ± SD from three independent experiments, with individual data points superimposed. Statistical analysis was performed using one-way ANOVA with Tukey's post-hoc test for multiple comparisons. *p < 0.05, **p < 0.01, ***p < 0.001, ****p < 0.0001. (I, J) Immunofluorescence analysis of γH2AX foci in the indicated EC cells treated with cisplatin (5 µM) for 24 hours. Nuclei were stained with DAPI (blue). (I) Representative immunofluorescence images. Scale bar = 20 µm. (J) Quantification of γH2AX foci-positive cells. Data are presented as mean ± SD from three independent experiments, with individual data points superimposed. Statistical analysis was performed using one-way ANOVA with Tukey's post-hoc test for multiple comparisons. *p < 0.05, **p < 0.01, ***p < 0.001, ****p < 0.0001. (K) Western blot analysis of RBM39, RAD50, γH2AX, and cleaved caspase-3 protein levels in the indicated EC cells treated with cisplatin (5 µM) for 48 hours. (L-Q) Densitometric quantification of , RAD50(L, M), γH2AX(N, O) and cleaved caspase-3(P, Q) protein levels from (K). Band intensities were quantified using ImageJ software and normalized to β-actin as a loading control. Data are presented as mean ± SD from three independent experiments, with individual data points superimposed. Statistical analysis was performed using one-way ANOVA with Tukey's post-hoc test for multiple comparisons. *p < 0.05, **p < 0.01, ***p < 0.001, ****p < 0.0001. (R, S) Alkaline comet assay performed in the indicated EC cells treated with cisplatin (5 µM) for 48 hours. (R) Representative images. Scale bar = 20 µm. (S) Quantification of comet tail moment. Data are presented as mean ± SD from three independent experiments, with individual data points superimposed. Statistical analysis was performed using one-way ANOVA with Tukey's post-hoc test for multiple comparisons. *p < 0.05, **p < 0.01, ***p < 0.001, ****p < 0.0001.

**Fig. 6:Functional validation of RBM39 phosphorylation at Ser117 in CDK13‑mediated cisplatin resistance (related to Figure 5).**

1. Densitometric quantification of p-RBM39 levels from Figure 5B. Band intensities were quantified using ImageJ software and normalized to total RBM39 as a loading control. Data are presented as mean ± SD from three independent experiments, with individual data points superimposed. Statistical analysis was performed using unpaired two-tailed Student's t-test. *p < 0.05, **p < 0.01, ***p < 0.001, ****p < 0.0001. (B) Densitometric quantification of p-RBM39 levels from Figure 5C. Cells were treated with or without 1NM-PP1 (10 µM) as indicated. p-RBM39 signal intensity was normalized to total RBM39 as a loading control. Data are presented as mean ± SD from three independent experiments, with individual data points superimposed. Statistical analysis was performed using unpaired two-tailed Student's t-test. *p < 0.05, **p < 0.01, ***p < 0.001, ****p < 0.0001. (C) Mass spectrometric analysis of Ishikawa cells transfected with shCDK13 or shNC identified a significant reduction in phosphorylation at the S117 residue of RBM39 upon CDK13 knockdown. (D) Densitometric quantification of p-RBM39 levels from Figure 5D. Band intensities were quantified using ImageJ software and normalized to total RBM39 as a loading control. Data are presented as mean ± SD from three independent experiments, with individual data points superimposed. Statistical analysis was performed using unpaired two-tailed Student's t-test. *p < 0.05, **p < 0.01, ***p < 0.001, ****p < 0.0001. (E) Densitometric quantification of RAD50 protein levels from Figure 5D. Band intensities were quantified using ImageJ software and normalized to β-actin as a loading control. Data are presented as mean ± SD from three independent experiments, with individual data points superimposed. Statistical analysis was performed using unpaired two-tailed Student's t-test. *p < 0.05, **p < 0.01, ***p < 0.001, ****p < 0.0001. (F) Densitometric quantification of p-RBM39 levels from Figure 5E. Band intensities were quantified using ImageJ software and normalized to total RBM39 as a loading control. Data are presented as mean ± SD from three independent experiments, with individual data points superimposed. Statistical analysis was performed using unpaired two-tailed Student's t-test. *p < 0.05, **p < 0.01, ***p < 0.001, ****p < 0.0001. (G, H) Colony formation assays were performed in Ishikawa and AN3CA cells co-transfected with the indicated constructs and treated with increasing concentrations of cisplatin. Quantification is shown in (H). Data are presented as mean ± SD from three independent experiments, with individual data points superimposed. Statistical analysis was performed using one-way ANOVA with Tukey's post-hoc test for multiple comparisons. ***p < 0.001, ****p < 0.0001. (I, J) Immunofluorescence analysis of γH2AX foci in the indicated EC cells treated with cisplatin (5 µM) for 24 hours. Nuclei were stained with DAPI (blue). (I) Representative immunofluorescence images. Scale bar = 20 µm. (J) Quantification of γH2AX foci-positive cells. Data are presented as mean ± SD from three independent experiments, with individual data points superimposed. Statistical analysis was performed using one-way ANOVA with Tukey's post-hoc test for multiple comparisons. *p < 0.05, **p < 0.01, ***p < 0.001, ****p < 0.0001.

**Fig. 7:Phospho-mimetic RBM39 S117D rescues CDK13 knockdown-induced defects in DNA damage repair and cisplatin resistance (related to Figure 5).**

(A) Under CDK13 knockdown conditions, Western blot analysis was performed to detect CDK13, RBM39, RAD50, γH2AX, cleaved caspase-3, and β-actin in Ishikawa cells transfected with vector, RBM39 WT, or the phospho-mimetic mutant RBM39 S117D.

(B) Densitometric quantification of RAD50, γ-H2AX and cleaved caspase-3 protein levels from (A). Band intensities were quantified using ImageJ software and normalized to β-actin as a loading control. Data are presented as mean ± SD from three independent experiments, with individual data points superimposed. Statistical analysis was performed using unpaired two-tailed Student's t-test. *p < 0.05, **p < 0.01, ***p < 0.001, ****p < 0.0001. (C) Under CDK13 knockdown conditions, Western blot analysis was performed to detect CDK13, RBM39, RAD50, γH2AX, cleaved caspase-3, and β-actin in ANC3A cells transfected with vector, RBM39 WT, or the phospho-mimetic mutant RBM39 S117D. (D) Densitometric quantification of RAD50 γ-H2AX and cleaved caspase-3 protein levels from (C). Quantification was performed as described in (B). Data are presented as mean ± SD from three independent experiments, with individual data points superimposed. Statistical analysis was performed using unpaired two-tailed Student's t-test. *p < 0.05, **p < 0.01, ***p < 0.001, ****p < 0.0001. (E, F) Alkaline comet assays. Cells were treated with cisplatin for 48 h. (E) Representative images of comet tails. Scale bar = 20 µm. (F) Quantification of tail DNA percentage. Data are presented as mean ± SD from three independent experiments, with individual data points superimposed. Statistical analysis was performed using one-way ANOVA with Tukey's post-hoc test for multiple comparisons. *p < 0.05, **p < 0.01, ***p < 0.001, ****p < 0.0001. (G, H) Colony formation assays. Cells were cultured with cisplatin for 14 days. (G) Representative images of colonies. (H) Quantification of relative colony survival. Data are presented as mean ± SD from three independent experiments, with individual data points superimposed. Statistical analysis was performed using one-way ANOVA with Tukey's post-hoc test for multiple comparisons. *p < 0.05, **p < 0.01, ***p < 0.001, ****p < 0.0001.

**Fig. 8:RBM39 binds to *RAD50* mRNA and enhances its stability in a phosphorylation‑dependent manner (related to Figure 6).**

(A) *RAD50* mRNA levels measured by RT-qPCR in Ishikawa and AN3CA cells following RBM39 knockdown. Data are presented as mean ± SD from three independent experiments, with individual data points superimposed. Statistical analysis was performed using unpaired two-tailed Student's t-test. **p < 0.01, ***p < 0.001, ****p < 0.0001 vs. siNC. (B) RNA pull-down assay using biotinylated probes for OGG1 (positive control) and RAD50, followed by Western blot detection of bound RBM39 protein in Ishikawa and AN3CA cell lysates. Representative images from three independent experiments are shown. (C) Co-IP analysis using HA antibody in Ishikawa cells transfected with HA-RBM39, validating successful immunoprecipitation. Representative images from three independent experiments are shown. (D) RIP assay using HA antibody in Ishikawa cells transfected with HA-RBM39, followed by RT-qPCR analysis of enriched *OGG1*, *RAD50*, and *GAPDH* mRNAs. Data are presented as mean ± SD from three independent experiments, with individual data points superimposed. Statistical analysis was performed using one-way ANOVA with Tukey's post-hoc test for multiple comparisons. ****p < 0.0001 vs. GAPDH. (E, F) *RAD50* mRNA stability in Ishikawa (E) and AN3CA (F) cells transfected with HA-RBM39 WT or HA-RBM39 S117A and treated with Actinomycin D (Act D, 5 µg/mL). Data are presented as mean ± SD from three independent experiments, with individual data points superimposed. Statistical analysis was performed using two-way ANOVA with Tukey's post-hoc test for multiple comparisons. **p < 0.01, ***p < 0.001 vs. RBM39-WT.

**Fig. 9:Schematic model of the CDK13/RBM39/RAD50 signaling axis in promoting cisplatin resistance in endometrial cancer (related to Figures 1-6).**

Proposed model illustrating the CDK13/RBM39/RAD50 signaling axis in promoting DNA damage repair and mediating platinum resistance in EC cells. The consequent augmentation of DNA damage repair capacity promotes cisplatin resistance in tumor cells.
